# Aroma precursors of cigars from different tobacco parts and origins, and their correlations with sensory characteristics

**DOI:** 10.3389/fpls.2023.1264739

**Published:** 2023-12-13

**Authors:** Zhaoliang Geng, Pei He, Huajun Gao, Jian Liu, Jun Qiu, Bin Cai

**Affiliations:** ^1^ Haikou Cigar Research Institute, Hainan Provincial Branch of China National Tobacco Corporation, Haikou, China; ^2^ Institute of Tobacco Research, Chinese Academy of Agricultural Sciences, Qingdao, China; ^3^ Workshop of Cut Tobacco Production, Hainan Hongta Cigarette Company Limited, Haikou, China; ^4^ Department of Chemistry, Beijing Life Science Academy, Beijing, China

**Keywords:** cigar, aroma precursor, sensory, tobacco part, origin

## Abstract

Cigars are developing rapidly around the world, but the content characteristics of aroma precursors and their contribution to sensory perception have not been fully elucidated. In this study, 69 aroma precursors from 61 tobaccos of different parts and origins were systematically determined, and the sensory characteristics of middle leaves from different origins and their correlation with aroma precursors were evaluated. The results showed that tobacco parts mainly affected amino acid content, and contents of nicotine, oxalic acid, malic acid, isovaleric acid, cystine, glutarnine, glycine, isoleucine, glutamicacid, asparticacid, and fructose-proline were significantly changed. Tobacco origins mainly influenced the contents of amino acids, polyacids and high fatty acids, and sugar alcohols, and significantly affected the contents of myosmine, anabasine, nonanoic acid, propanetriol, mannitol, mannose, glucose, alanine, arginine, glutarnine, glutamicacid, histidine, serine, threonine, tryptophan, fructose-alanine, and fructose-asparagine. The flavor characteristics were prominent by wood aroma, and the style and quality characteristics varied greatly among different origins of middle leaves. There were 34, 21, and 22 aroma precursors with high correlations with flavor, style, and quality characteristics. This study provides support for regulating the content and coordination of aroma precursors in different tobacco parts and origins to improve sensory characteristics.

## 1 Introduction

The core of a cigar is the filler, which determines the style and quality of the cigar. High-quality cigars require a filler with typical cigar style manifestation, and sensory characteristics such as good aroma quality, rich aroma volume, mellow eating flavor, rich and varied smoke, low irritation, and fewer impurities. Therefore, the chemical composition of filler tobacco is an important factor affecting the taste and quality of cigars (Sun H, et al., 2020). The cigar has shown rapid development in recent years, but there is still a large gap between the common and high-quality tobacco in many production areas, and the lack of high-quality tobacco has become an obstacle to the development of the cigar industry.

The sensory quality determines the industrial availability of tobacco, and the content and composition of the chemical components and their coordination directly affect the sensory quality of tobacco (Yan et al., 2021). Aroma precursors are generally stable and odorless, but they can be subjected to a series of reactions during cigarette smoking to produce volatile small-molecule aldehydes, ketones, acids, esters, furans, pyrazines, and other substances with different odor characteristics, which have positive effects on improving the aroma, odor, and color quality of tobacco products. In the process of tobacco aroma formation, only about 30% of volatile components from the tobacco are directly transferred to the smoke, while the transformation of non-volatile precursors into various complex reactions such as pyrolysis, polymerization, and condensation is the key basis for the formation of tobacco smoke (Banožić et al., 2020).

The composition and content of alkaloids directly affect the strength, stimulation, taste, and safety of tobacco products (Li et al., 2001). Organic acids in tobacco include volatile lower fatty acids, semi-volatile higher fatty acids, and non-volatile polyacids. The higher fatty acids can enhance the fatty or spicy flavor of the tobacco (Li et al., 2021). Polyacids cannot act directly on the smoke, but can improve the eating taste and enhance the sensory comfort of cigarettes by regulating the pH of the smoke (Shen et al., 2018; Piao et al., 2022). Volatile acids enter the smoke directly when the cigarette is smoked and have a significant effect on the taste (Gao F, et al., 2021). Sugars play an important role in balancing nitrogenous compounds and improving the smoke strength and irritation (Wang et al., 2021), and sugar alcohols of natural origin, such as inositol in tobacco, can reduce the smoke irritation and improve the smoking quality of cigarettes (Seeman et al., 2003). The amino acids in tobacco are closely related to the aroma and taste, and the appropriate amount can help improve the strength and fullness of the smoke, while too much can increase the irritation and bitterness (Deng et al., 2011). In the curing and aging process of tobacco, amino acids and reducing sugars undergo a Maillard reaction to produce important aroma substances (Wahlberg et al., 1977), and the intermediate product-Amadori compounds do not show aroma themselves, but after heating, rearrangement, dehydration and cleavage occur to produce a large number of flavor substances that give tobacco the unique aroma (Kanzler et al., 2017).

Current studies on the chemical composition of cigar tobacco have focused on the stages of harvesting, curing, fermentation, and the analysis of the chemical composition and sensory quality of cigar tobacco after fermentation in different production areas and varieties. Gao S, et al. (2021) analyzed the differences in conventional chemical composition and latent aroma substances carotenoids and chlorophyll in GH1 and GH2 tobacco from Shifang and Liangshan. Sun Y, et al. (2020) found that the conversion of nicotine to nornicotine was prevalent in cigar tobacco. Xu et al. (2019) determined the conventional chemical composition and polyphenol content of 50 cigar tobaccos and found that the chemical composition was closely related to the sensory quality. Zheng et al. (2022) analyzed the style characteristics of cigar tobacco from different origins, including Dominica, Indonesia, and the United States, and found that Dominica had the best quality with rich aroma, good aroma quality, and less impurities. Sensory characteristics are the core content of the tobacco product quality, which is reflected by the overall characteristics of the tobacco chemical composition (Ma et al., 2018). Right now, the studies on the chemical composition of cigar tobacco are limited to a few constituents, while the chemical composition of tobacco is quite complex, and it is difficult to comprehensively reflect the cigar products quality only through certain constituents in the tobacco (Hu et al., 2001). Therefore, it is necessary to build the core technology of cigar products based on the rich aroma precursors as the investigation object, which truly reflects the influence of process conditions on product quality.

In this study, seven categories of 69 reported important aroma precursors, including alkaloids, polyacids and higher fatty acids, volatile acids, amino acids, sugar alcohols, Amadori compounds, and polyphenols (**Table 1**), were systematically determined in 61 cigar tobaccos, the aroma precursors differences between upper and middle leaves among different tobacco origins, varieties, and production years were compared, and the aroma precursors in different origins among various tobacco parts, varieties, and production years were also evaluated, and the flavor, style, and quality characteristics of middle leaves from different origins and their correlation with aroma precursors were analyzed. It provided a basis for the sensory control of cigars and the improvement of tobacco quality.

**Table 1:** Varieties of aroma precursors of cigar tobaccos.

No.	Alkaloids	Polyacids and higher fatty acids	Volatile acids	Sugar alcohols	Amino acids		Amadori compounds	Polyphenols
1	Nicotine (A1)	Oxalic acid (PF1)	Acetic acid (V1)	Propanetriol (S1)	Alanine (Ala/AC1)	Leucine (Leu/AC11)	Fructose-Ala (Fru-Ala /AM1)	Neochlorogenic acid (P1)
2	Nornicotine (A2)	Malonic acid (PF2)	Formic acid (V2)	Mannitol (S2)	Arginine (Arg/AC2)	Lysine (Lys/AC12)	Fructose-Asn (Fru-Asn /AM2)	Chlorogenic acid (P2)
3	Myosmine (A3)	Succinic acid (PF3)	Propionic acid (V3)	Mannose (S3)	Asparagine (Asn/AC3)	Phenylalanine (Phe/AC13)	Fructose-Glu (Fru-Glu /AM3)	Cryptochlorogenic acid (P3)
4	Anabasine (A4)	Malic acid (PF4)	Isobutyric acid (V4)	Fructose (S4)	Asparticacid (Asp/AC4)	Proline (Pro/AC14)	Fructose-Gly (Fru-Gly /AM4)	Scopoletin (P4)
5	Anatabine (A5)	Palmitic acid (PF5)	Butyric acid (V5)	Inositol (S5)	Cystine (Cys/AC5)	Serine (Ser/AC15)	Fructose-Leu (Fru-Leu /AM5)	Rutin (P5)
6		Citric acid (PF6)	Isovaleric acid (V6)	Xylitol (S6)	Glutarnine (Gln/AC6)	Threonine (Thr/AC16)	Fructose-Ile (Fru-Ile /AM6)	
7		Oleic acid (PF7)	Valeric acid (V7)	Raffinose (S7)	Glutamicacid (Glu/AC7)	Tryptophan (Trp/AC17)	Fructose-Phe (Fru-Phe /AM7)	
8		Linoleic acid (PF8)	Hexanoic acid (V8)	Glucose (S8)	Glycine (Gly/AC8)	Tyrosine (Tyr/AC18)	Fructose-Pro (Fru-Pro /AM8)	
9		Linolenic acid (PF9)	Octanoic acid (V9)	Rhamnose (S9)	Histidine (His/AC9)	Valine (Val/AC19)	Fructose-Trp (Fru-Trp /AM9)	
10			Nonanoic acid (V10)	Sucrose (S10)	Isoleucine (Ile/AC10)		Fructose-Val (Fru-Val /AM10)	
11							GLU (Glucosamine/AM11)	

## 2 Materials and methods

### 2.1 Materials and reagents

Cigar tobacco samples were collected from the production areas of Guangcun, Changjiang, Baisha, Datian, Wuzhishan, and Chengmai in Hainan Province, China (**Table S1**). The standards of Amadori compounds, adipic acid, and phenethyl propionate were purchased from Anpel Laboratory Technologies (Shanghai) Inc. (Shanghai, China), the standards of alkaloids, sugar alcohols, amino acids, polyphenols, and other chemicals were purchased from Sinopharm Chemical Reagent Co., Ltd (Shanghai, China), nicotine and Fru-Gly were purchased from Sigma-Aldrich Co. (St. Louis, MO, USA) and Toronto Research Chemicals Inc. (Toronto, Canada), respectively.

### 2.2 Determination of aroma precursors

#### 2.2.1 Alkaloids analysis

Two milliliters of 5% NaOH solution and 0.3 g of sample were placed in a 50 mL centrifuge tube, the tube was gently shaken to wet the sample and held for 15 min. One milliliter of primary internal standard solution and 20 mL of extraction solution were added to the tube, the tube was sealed and shaken for 20 min, then centrifuged at 3000 rpm for 5 min to separate the sample from the extractant. Four grams anhydrous Na_2_SO_4_ was added into a sterile syringe, and 2 mL lower solution was added into the syringe, then the filtrate was collected into a 2 mL chromatography bottle.

The samples were detected by gas chromatography-mass spectrometry (GC-MS, 7890B-7000C, Agilent Technologies Inc., Santa Clara, CA, USA) combined with a quartz capillary column (DB-5, 30 m×0.25 mm×0.25 μm, Agilent Technologies Inc.). The temperature program was as follows: the initial temperature was 100 °C and held for 3 min, then increased to 260 °C at a rate of 8 °C/min and held for 10 min. The carrier gas was He and the flow rate was 1.0 mL/min. The temperatures of the inlet, ion source, and transmission line were 250 °C, 230 °C, and 280 °C, respectively. The ionization voltage was 70 eV, and the scanning mode was selected ion monitoring (SIM).

#### 2.2.2 Polyacids and higher fatty acids analysis

One gram sample, 25 mL 10% (v/v) H_2_SO_4_-CH_3_OH solution, and 500 μL internal standard solution were mixed in a 100 mL conical flask, the flask was shaken at low speed overnight for the methyl esterification reaction, then the mixture was filtered through filter paper into a 250 mL separatory funnel containing 50 mL distilled water. The filtrate was extracted with 15 mL of dichloromethane, the extract was dried with baked anhydrous Na_2_SO_4_, and the anhydrous Na_2_SO_4_ was washed with 10 mL of dichloromethane. The three extracts were combined and filtered through a 0.45 μm nylon membrane.

The samples were detected by GC (6890N, Agilent Technologies Inc.) equipped with HP-FFAP (50.0 m×32. 0 μm×0.50 μm, Agilent Technologies Inc.). The temperature program was as follows: the initial temperature was 80 °C, increased to 150 °C at a rate of 10 °C/min, then increased to 220 °C at a rate of 8 °C/min and held for 15 min. The inlet and detector temperatures were 260 °C and 270 °C, respectively. The carrier gas was N_2_ at a flow rate of 1.8 mL/min, combustion gases were air and H_2_, with flow rates of 400 mL/min and 30 mL/min, respectively, and tail gas (N_2_) at a rate of 30 mL/min, the split ratio was 10:1, and the injection volume was 1 μL. Flame ionization was used as the detector.

#### 2.2.3 Volatile acid analysis

Ten grams sample, 15.0 mL acetone, and 150 μL internal standard extraction solution were added to a 30 mL glass tube, the tube was shaken at 1000 rpm for 30 min. After standing, the supernatant was separated and passed through a 0.45 μm organic phase filter membrane.

The samples were detected by GC-MS (7890A/5975C, Agilent Technologies Inc.) combined with a quartz capillary column (30 m×0.25 mm×0.25 μm). The temperature program was as follows: initial temperature was 50 °C and held for 2 min, increased to 190 °C at a rate of 4 °C/min, then increased to 240 °C and held for 10 min. The carrier gas was He and the flow rate was 1.0 mL/min. The split ratio was 10:1 and the injection volume was 2 μL. The inlet, ion source, and transmission line temperatures were 250 °C, 230 °C, and 240 °C, respectively. The ionization voltage was 70 eV and the scanning mode was SIM.

#### 2.2.4 Sugar alcohols and amino acids analysis

Ten milliliters 50% aqueous acetonitrile and 0.1 g sample were mixed in a 50 mL centrifuge tube, and the sample was extracted in a vortexer for 30 min, then the mixture was centrifuged at 4000 rpm for 5 min, and the supernatant was filtered by membrane. The samples were detected by liquid chromatograph-mass spectrometer (LC-MS, ACQUITY UPLC (QTRAP 4500), Waters Corporation, Milford, MA, USA). The determination of sugar alcohols was based on the method of Xu et al. (2011). For the determination of amino acids, the mobile phases were 0.2% triethylamine acetonitrile solution (A) and 0.2% triethylamine aqueous solution (B), and the injection volume was 2 μL. The gradient elution program was as follows: 0-0.5 min 95% A, 0.5-10 min 95% A-45% A, 10.5-13.0 min 95% A.

#### 2.2.5 Amadori compounds analysis

Twenty milliliters 30:70 (v/v) methanol/water solution and 0.1 g sample were mixed in a 50 mL centrifuge tube, the tube was vortexed for 10 min and the mixture was centrifuged at 5000 rpm for 10 min, then the supernatant was passed through a 0.45 μm membrane.

The samples were detected by LC-MS (ACQUITY UPLC (QTRAP 4500), Waters Corporation) equipped with ACE Excel C18-Amide column (2.1 mm×100 mm×5 μm, VWR (Shanghai) Co., Ltd, Shanghai, China). The mobile phases were 2% formic acid solution (A) and acetonitrile (B), the flow rate was 350 μL/min, the column temperature was 30 °C, and the injection volume was 2 μL. The gradient elution program was as follows: 0-0.1 min 89.5% A-78.0% A, 0.1-10.0 min 78.0% A. The ion source was electrospray ion source (ESI), the scan mode was positive ion scan, the detection mode was multiple reaction monitoring (MRM), and the ion source temperature was 550°C.

#### 2.2.6 Polyphenols analysis

Ten milliliters 50:50 (v/v) methanol/water solution and 0.5 g sample were mixed in a 50 mL centrifuge tube, the tube was vortexed for 30 min, and the extract was passed through a 0.45 μm aqueous phase membrane.

The samples were detected by high performance liquid chromatography (HPLC, 2695, Waters Corporation). The mobile phases were methanol (A) and 1% acetic acid solution (B), the flow rate was 400 μL/min, the injection volume was 5 μL, the column temperature was 30 °C, and the detection and reference wavelengths were 340 nm and 480 nm, respectively. The gradient elution program was as follows: 0-0.5 min 5% A-10% A, 0.5-11.6 min 10% A-100% A, 11.7-13.5 min 5% A.

### 2.3 Sensory evaluation

Due to the chemical coordination and sensory characteristics of middle leaf were the most suitable for the industrial availability, so the middle leaves were chosen for sensory analysis. Sensory evaluation was carried out by China Tobacco Hubei Industrial Co., Ltd. The sample moisture was balanced before smoking according to GB/T 16447 (Standardization Administration of China, 2004), and the flavor, style, and quality characteristics of the tobacco were quantitatively rated using the 0-5 equidistant scales (scales: 0-1: no to slightly significant, 2-3: moderately significant to still significant, 4-5: relatively significant to significant). Flavor characteristics included nutty, bean, coffee, woody, fruity, honey-sweet, floral, milk, resin, and roasted aromas. Style characteristics included undesirable aromas (protein, green, and burnt), taste (bitterness, sweetness, spicy, and astringent), smoke concentration, smoke strength, transmissibility, mellowness, and cigar-style manifestation. The quality characteristics include aroma quality, aroma volume, irritation, combustibility, gray, miscellaneous aroma, aftertaste, and sweetness.

### 2.4 Statistical analysis

The results were expressed as mean ± standard deviation. The significance of the differences between the means of the samples was performed by one-way analysis of variance (ANOVA) Duncan’s multiple comparisons using IBM SPSS Statistics 25 (IBM Corp., Armonk, NY, USA), and *P*<0.05 was considered significant. Aroma precursors differences between upper and middle leaves were compared among different tobacco varieties, origins, and production years, the differences in origins were evaluated among various tobacco varieties, parts, and production years, and sensory evaluation was by the middle leaves of different origins among various tobacco varieties and production years. Partial least square-discrimination analysis (PLS-DA) was employed to correlate aroma precursors with different tobacco parts and origins, and to reveal the differential metabolites using Simca 14.1 (Mks Umetrics Ab Corp., Malmö, Sweden). The heatmap cluster was carried out through the OmicShare tool (https://www.omicshare.com/tools). Pearson correlation analysis was performed by Origin 2021b (OriginLab Corp., Northampton, MA, USA), and aroma precursors with |r|≥0.4 and *P*<0.05 were chosen for analysis.

## 3 Results and discussion

### 3.1 Effect of tobacco parts on aroma precursors

**Figure 1:**
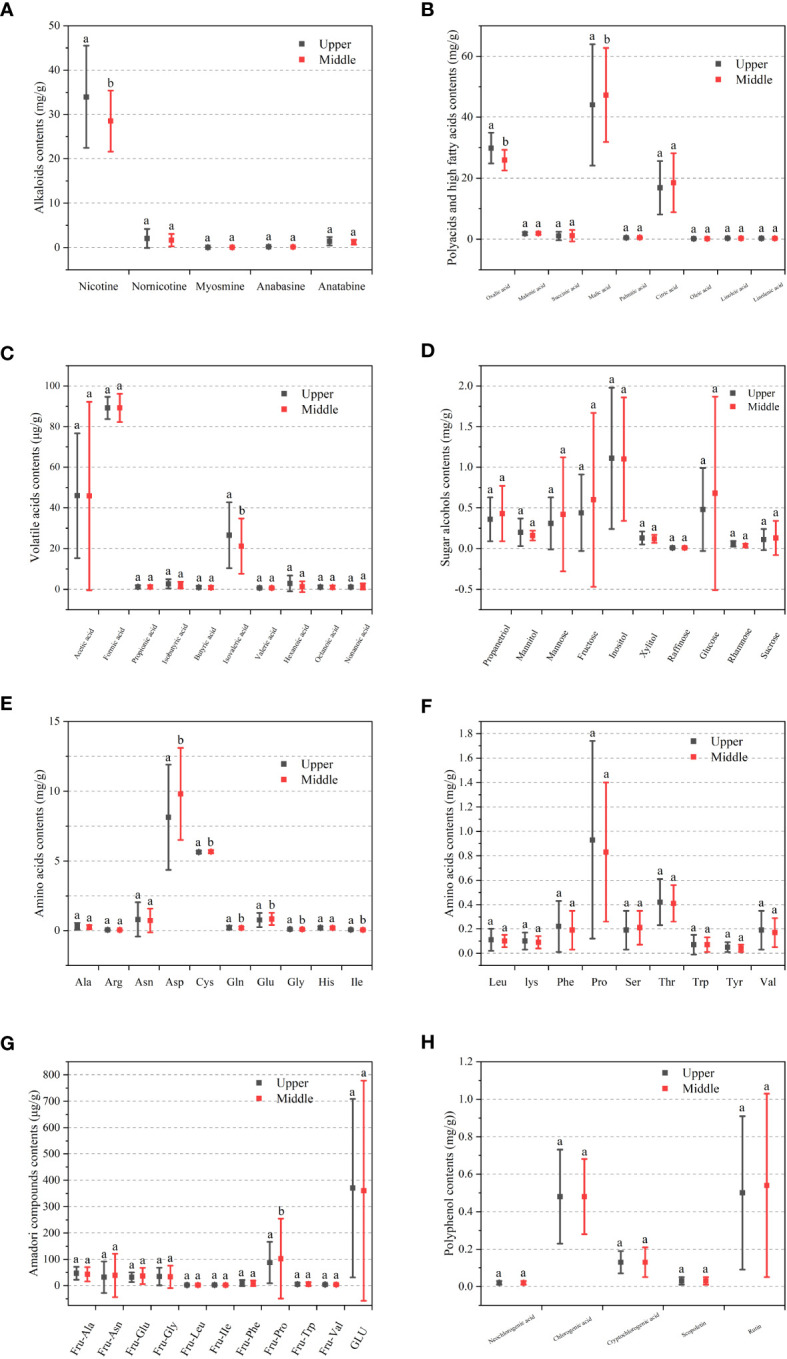
The contents of aroma precursors in cigar tobaccos of upper and middle parts. **(A)** Alkaloids. **(B)** Polyacids and higher fatty acids. **(C)** Volatile acids. **(D)** Amino acids. **(E)** Other amino acids. **(F)** Sugar alcohols. **(G)** Amadori compounds. **(H)** Polyphenols.

The nicotine and total alkaloid contents of the upper leaves were significantly higher than those of the middle leaves (**Figure 1A** and **Table S2**). Similar to flue-cured tobacco, the higher the part of the plant growth process, the higher the alkaloid content followed. The nicotine content was higher in both tobacco parts, and nornicotine, anatabine, and myosmine were lower, with 33.97 mg/L, 2.07 mg/L, 1.44 mg/L, and 0.07 mg/L in the upper leaves and 28.52 mg/L, 1.66 mg/L, 1.20 mg/L and 0.07 mg/L in the middle leaves, and nicotine accounted for 90.10% of the total alkaloids. The variations of total alkaloids and nicotine were large, and those of myosmine, anabasine, and anatabine were small. The variation coefficients of nornicotine, myosmine, and anatabine were large, with the highest of 102.90% and 84.34% for nicotine in the upper and middle leaves, respectively.

The total nicotine conversion rate of cigar tobacco was 6.59%, and the upper leaves were higher than the middle leaves, with the highest coefficients of 54.04% and 31.67%, respectively. Compared to flue-cured tobacco, cigars had low nicotine content and high nornicotine and nicotine conversion rates. The conversion of nicotine to nornicotine in cigar tobacco may be related to the curing and the number and stability of conversion sites (Tian et al., 2021). The coordination of nicotine with secondary alkaloids directly affects the physiological strength of tobacco products, and plants with high nicotine conversion are more likely to produce more tobacco-specific nitrosamines during curing and aging (Shi et al., 2008). 2019 Hainan 3 upper leaves from Changjiang had the highest nornicotine content of 54% nicotine conversion, and four other samples had more than 30% nicotine conversion rate. Adequate nicotine concentration can provide smokers with proper physiological stimulation, pleasant aroma, and mellow eating taste, and the formation of nornicotine is the result of nicotine demethylation, which has a negative effect on the smoke (Sun, 2010).

Oxalic acid content was significantly higher and malic acid content was lower in the upper leaves compared to the middle leaves (**Figure 1B** and **Table S3**). The highest malic acid content was found in both tobacco parts, followed by oxalic acid and citric acid, and oleic acid was the lowest, with 44.04 mg/g, 30.03 mg/g, 16.86 mg/g, and 0.21 mg/g in the upper leaves and 47.29 mg/g, 25.91 mg/g, 18.50 mg/g, and 0.19 mg/g in the middle leaves. The variation of palmitic acid was small in the upper leaves, and the variation coefficients of linolenic acid, citric acid, malic acid, linoleic acid, and palmitic acid were smaller.

The total polyacid and higher fatty acid contents of cigar tobacco were lower than those of flue-cured tobacco but they were all dominated by oxalic acid, malic acid, and citric acid, while citric acid, oxalic acid, and malic acid were dominated in burley. Tobacco nicotine and organic acids are often present in the form of bound organic salts, malate and oxalate can give a slightly flue-cured tobacco aroma, while citrate gives a sun-cured aroma. Hainan cigar tobacco tended to move closer to the flue-cured tobacco aroma, and the citric acid content can be increased accordingly to promote the cigar to change to the sun-cured aroma (Liu et al., 2002).

Compared with polyacids and higher fatty acids, the volatile acids content was low (**Figure 1C** and **Table S4**). The contents of total volatile acid and isovaleric acid of the upper leaves were significantly higher than those of the middle leaves, with 172.45 μg/g and 26.55 μg/g for the former and 164.93 μg/g and 21.19 μg/g for the latter. The formic acid content was the highest in the two tobacco parts (89.26 μg/g), accounting for 52.89% of the total volatile acids, followed by acetic acid (45.94 μg/g) and isovaleric acid (23.91 μg/g), and valeric acid was the lowest (0.72 μg/g). The variation of acetic acid was large, followed by isovaleric acid and formic acid, and valeric acid was small. Hexanoic acid had the largest variation coefficient and formic acid had the smallest. The dominant volatile acids were the same in cigar and flue-cured tobacco, but formic acid was higher in the former, followed by acetic acid, and vice versa in the latter. The pH of cigar tobacco was determined by the volatile acid and alkaloid. Cigar tobacco has a higher alkaline and a lower acidic component, resulting in a higher pH than flue-cured tobacco.

Inositol content was the highest in the upper and middle leaves (**Figure 1D** and **Table S5**), similar to flue-cured tobacco (Wang, 2011), followed by glucose and fructose, and raffinose was the lowest. The variation coefficients for sucrose, fructose, glucose, and mannose were all large. The sugar alcohols in the mainstream smoke were directly converted from sugar alcohols in the tobacco leaves that were not involved in the reaction, which may affect the sweetness in the sensory evaluation of cigarettes.

The contents of acidic amino acids Glu and Asp decreased significantly in the upper leaves compared to the middle leaves (**Figures 1E, F** and **Table S6**), and the contents of neutral amino acids Cys, Gln, Gly, and Lle increased. Asp content was highest in both tobacco parts, followed by Pro and Asn in the upper leaves, and Glu and Pro in the middle leaves, and Cys was the lowest. The variation of Asp was the largest, followed by Asn and Pro, and those of Cys was the smallest. The variation coefficients of Cys, His, Leu, and Lys were smaller, and Asn, Phe, and Trp were larger. The dominant amino acids in the cigar, red-sun cured, and burley tobacco were all Asp, but the formation pathway was different, with fresh red sun-cured tobacco having the highest Asp content (Li et al., 2018), while the Asp of burley increased probably due to the curing process (Sun et al., 2014). Cigar tobacco had the highest proportion of acidic amino acids, followed by neutral and basic amino acids. The proportion of basic amino acids in flue-cured tobacco was lower than that in cigar tobacco, probably due to the basic amino acids such as His, Arg, and Lys in flue-cured tobacco having a higher activity in the Maillard reaction, and they were consumed to a high degree during the curing process (Kwak and Lim, 2004).

The Fru-Pro content of the upper leaves was significantly lower than that of the middle leaves (**Figure 1G** and **Table S7**), and the variation coefficient of Fru-Asn was larger in the middle leaves. GLU content was the highest in the two tobacco parts, accounting for 58.10% of the Amadori compounds, followed by Fru-Pro and Fru-Ala, and Fru-IIe and Fru-Leu were lower. GLU variation was the highest, followed by Fru-Pro, Fru-Asn. Fru-IIe, Fru-Leu, and Fru-Val were the least. The variation coefficients of Fru-IIe, Fru-Leu, and Fru-Trp were large. The varieties and contents of aroma substances produced by thermal cracking differed from the varieties and contents of Amadori compounds, which had different effects on the flavor and quality of flue-cured tobacco (Wang, 2021). The percentage of Amadori compounds content and the distribution of their corresponding amino acid content in the tobacco leaves were not consistent, indicating that there were differences in the activity of different free amino acids involved in the Maillard reaction, similar to flue-cured tobacco (Zhang et al., 2021).

Rutin content was the highest in the two tobacco parts (**Figure 1H** and **Table S8**), followed by chlorogenic acid, similar to flue-cured tobacco. Both accounted for 84.75% of the total polyphenol content, and neochlorogenic acid was lower. The variation of chlorogenic acid was large, and the variation coefficient of rutin was larger. The chlorogenic acid content of cigar tobacco after curing was higher than that of rutin (Xu et al., 2016), probably due to a larger decrease in chlorogenic acid content during fermentation.

**Figure 2:**
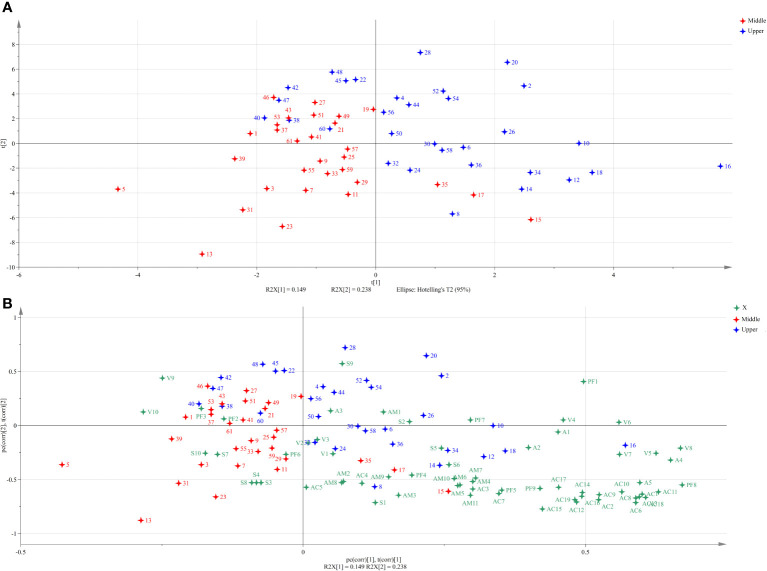
Score scatter plot **(A)** and biplot **(B)** for partial least square-discrimination analysis (PLS-DA) of aroma precursors in cigar tobaccos of upper and middle parts. The aroma precursors and samples used for analysis were listed in **Tables 1** and **S1**, respectively.

The results of PLS-DA were shown in **Figure 2A**. The upper and middle leaves were obviously separated based on the different aroma precursors. The middle leaves were distributed on the left side of the axis, and the upper leaves were mostly located on the right side of the axis. As shown in **Figure 2B**, the middle leaves were closely related to sucrose, raffinose, glucose, fructose, mannose, citric acid, malonic acid, and succinic acid, while the upper leaves were associated with rhamnose, mannitol, inositol, xylitol, oleic acid, isobutyric acid, propionic acid, formic acid, acetic acid, myosmine, nornicotine, nicotine, and Fru-Ala. Nicotine, anabasine, anatabine, oxalic acid, palmitic acid, linoleic acid, Ala, Asp, Cys, Glu, Gly, Ile, Leu, Ser, isobutyric acid, butyric acid, isovaleric acid, hexanoic acid, mannitol, and rhamnose (variable importance in the projection (VIP) >1) contributed greatly to distinguish the upper and middle leaves.

### 3.2 Effect of tobacco origin on aroma precursors

**Figure 3:**
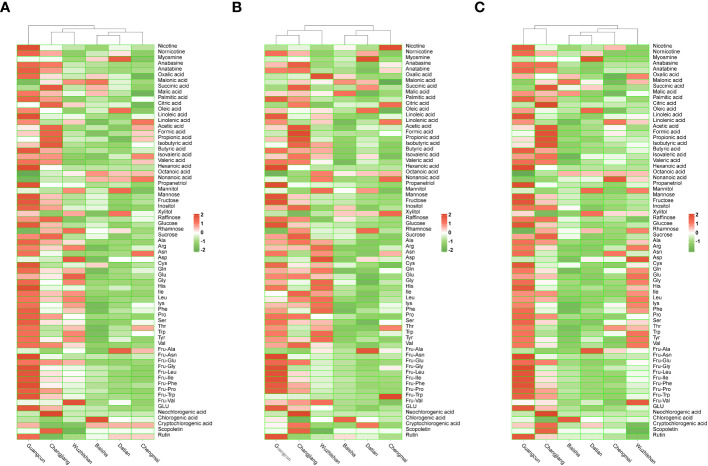
Heatmap cluster of aroma precursors in cigar tobaccos from Guangcun, Changjiang, Baisha, Datian, Wuzhishan, and Chengmai. **(A)** Upper leaves. **(B)** Middle leaves. **(C)** The mixtures of upper and middle leaves. The data was performed row normalization before cluster analysis.

There were significant differences in the content of myosmine and anabasine in different origins (**Figures 3A–C** and **Table S9**), and the myosmine content in Datian increased by 57.14-120.00% compared with other origins, and the anabasine content in the upper and middle leaves of Guangcun increased by 66.67-92.31% and 6.25-41.67%, respectively.

As shown in **Table S10**, significant differences were observed in the contents of total polyacids, succinic acid, malic acid, palmitic acid, oleic acid, and linoleic acid among different origins. Among them, the total polyacid content of the upper leaves of Guangcun, Changjiang, and Baisha was significantly higher, reaching 107.35 mg/g, 103.13 mg/g, and 96.08 mg/g, respectively. The contents of palmitic acid and linoleic acid in the upper leaves of Guangcun increased by 34.69-78.38% and 52.94-100.00%, and the contents of palmitic acid in the upper leaves and succinic acid of Changjiang increased by 0.66-3.61 and 0.18-0.57 times, respectively, while the content of malic acid in the middle leaves of Baisha increased by 14.86-88.08%. The total polyacid and malic acid of the upper leaves and the oleic acid of the upper leaves of Chengmai decreased significantly, the total volatile acid and malic acid of the middle leaves of Datian decreased, the oleic acid of the upper leaves of Wuzhishan decreased significantly. Fu et al. (2014) determined that the acid-base ratio of flue-cured tobacco was between 11 and 24, and the quality of flue-cured tobacco improved with the increase of acid-base ratio, but the acid-base ratio in this study was lower than 3.0, and the effect of the acid-base ratio of cigar tobacco on the quality needs to be studied.

The total volatile acid content of the upper leaves of Changjiang increased significantly compared with other origins (**Table S11**), reaching 201.92 μg/g, while that of Baisha, Datian, and Wuzhishan decreased significantly to 143.48 μg/g, 141.12 μg/g, and 140.80 μg/g, respectively. Formic acid, acetic acid, and isovaleric acid accounted for 94.29% of the total volatile acid. The content of nonanoic acid in the upper leaves of Guangcun decreased significantly (0.55 μg/g). The volatile acid content of cigar tobacco was higher than that of flue-cured tobacco (Gao F, et al., 2021), but inconsistent with what Zhang reported (Zhang et al., 2022), probably due to the large variation in volatile acid content of tobacco from different origins. The volatile acid content of tobacco is strongly influenced by maturity, and the total volatile acid is lower in unripe or overripe harvested tobacco after curing (Wang et al., 2007). The green and unripe of Datian and Chengmai were higher than other origins, but the total volatile acid content was low in Datian and medium in Chengmai, indicating that the volatile acid content of cigar tobacco was also related to soil conditions and fertilizers (Peng et al., 2014).

The contents of propanetriol, mannitol, mannose, and glucose were significantly different among different origins (**Table S12**). Among them, the glucose content increased by 0.87-8.77 times in the middle leaves of Guangcun, the propanetriol of the middle leaves and mannose of the upper leaves of Datian were significantly decreased, while the propanetriol of the middle leaves and mannitol of the upper leaves of Chengmai were decreased.

Significant differences were observed in the contents of Ala, Arg, Gln, Glu, His, Ser, Thr, and Trp among the different origins (**Table S13**). Among them, the contents of Arg, Gln, His, Ser, Thr, and Trp in the upper leaves of Guangcun were 0.50-2.0, 0.41-3.43, 0.56-0.87, 0.68-5.40, 0.14-1.32, and 0.10-4.50 times higher than those of other origins, and the contents of Arg, Gln, and Glu in the upper leaves of Wuzhishan were 0.50-2.0, 0.14-2.57, and 0.30-2.23 times, and the content of Arg in the upper leaves of Changjiang increased by 25-150%, while the content of Ala in the upper leaves of Chengmai decreased significantly. The differences in free amino acids significantly affected the quality of tobacco, and the differences in their composition and content caused the differences in Maillard reaction products and aroma and taste.

There were significant differences in Fru-Ala and Fru-Asn of Amadori compounds from different origins (**Table S14**), and the Fru-Ala content in middle leaves of Datian increased by 0.60-2.01 times, and Fru-Asn in upper and middle leaves of Guangcun increased by 2.41-9.51 times and 2.57-11.66 times, respectively. However, there were no differences in polyphenol contents among different origins (**Table S15**).

**Figure 4:**
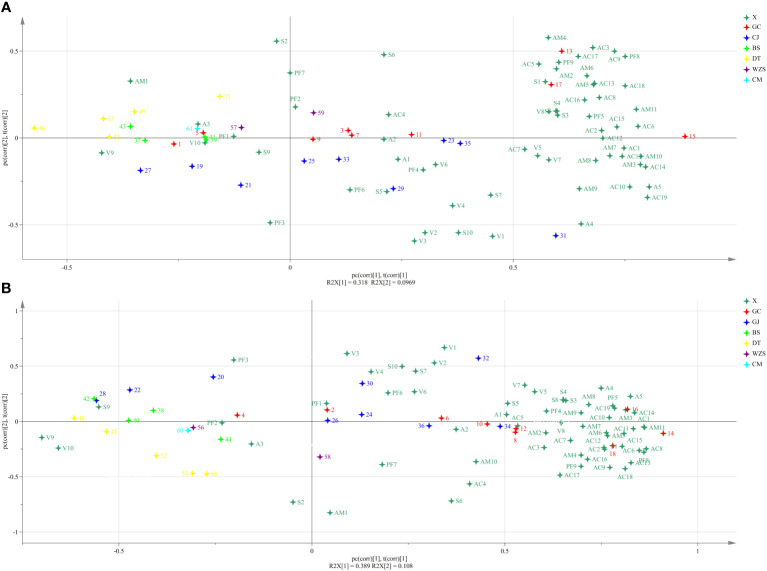
Biplot for partial least square-discrimination analysis (PLS-DA) of aroma precursors in cigar tobaccos of Guangcun (GC), Changjiang (CJ), Baisha (BS), Datian (DT), Wuzhishan (WZS), and Chengmai (CM). **(A)** Middle leaves. **(B)** Upper leaves. The aroma precursors and samples used for analysis were listed in **Tables 1** and **S1**, respectively.

As shown in **Figure 4A**, the middle leaves from different origins were separated apparently based on the PLS-DA. Among them, the middle leaves of Guangcun were related to nornicotine, hexanoic acid, palmitic acid, linolenic acid, Fru-Asn, Fru-Gly, Fru-Leu, Fru-Ile, Asn, Asp, Cys, Gly, Phe, Thr, and Trp, Changjiang was associated with nicotine, isovaleric acid, inositol, rhamnose, succinic acid, malic acid, and citric acid, Wuzhishan was related to oxalic acid, malonic acid, and oleic acid, while Datian was related to Fru-Ala. Anabasine, anatabine, succinic acid, palmitic acid, linoleic acid, linolenic acid, formic acid, propionic acid, isobutyric acid, hexanoic acid, propanetriol, mannitol, inositol, Fru-Ala, Fru-Asn, Fru-Gly, Asn, His, Ile, Phe, Pro, Ser, Thr, Trp, and Val (VIP >1) significantly contributed to differ the middle leaves from different origins. In addition, the upper leaves from different origins were also separated by the PLS-DA (**Figure 4B**), and anabasine, anatabine, succinic acid, palmitic acid, linoleic acid, linolenic acid, acetic acid, propionic acid, isobutyric acid, hexanoic acid, octanoic acid, nonanoic acid, propanetriol, mannitol, xylitol, Fru-Ala, Ala, Gly, His, Phe, Pro, Thr, and Trp (VIP >1) contributed to differing the upper leaves from different origins.

### 3.3 Sensory characteristics of middle leaves from different origins

**Figure 5:**
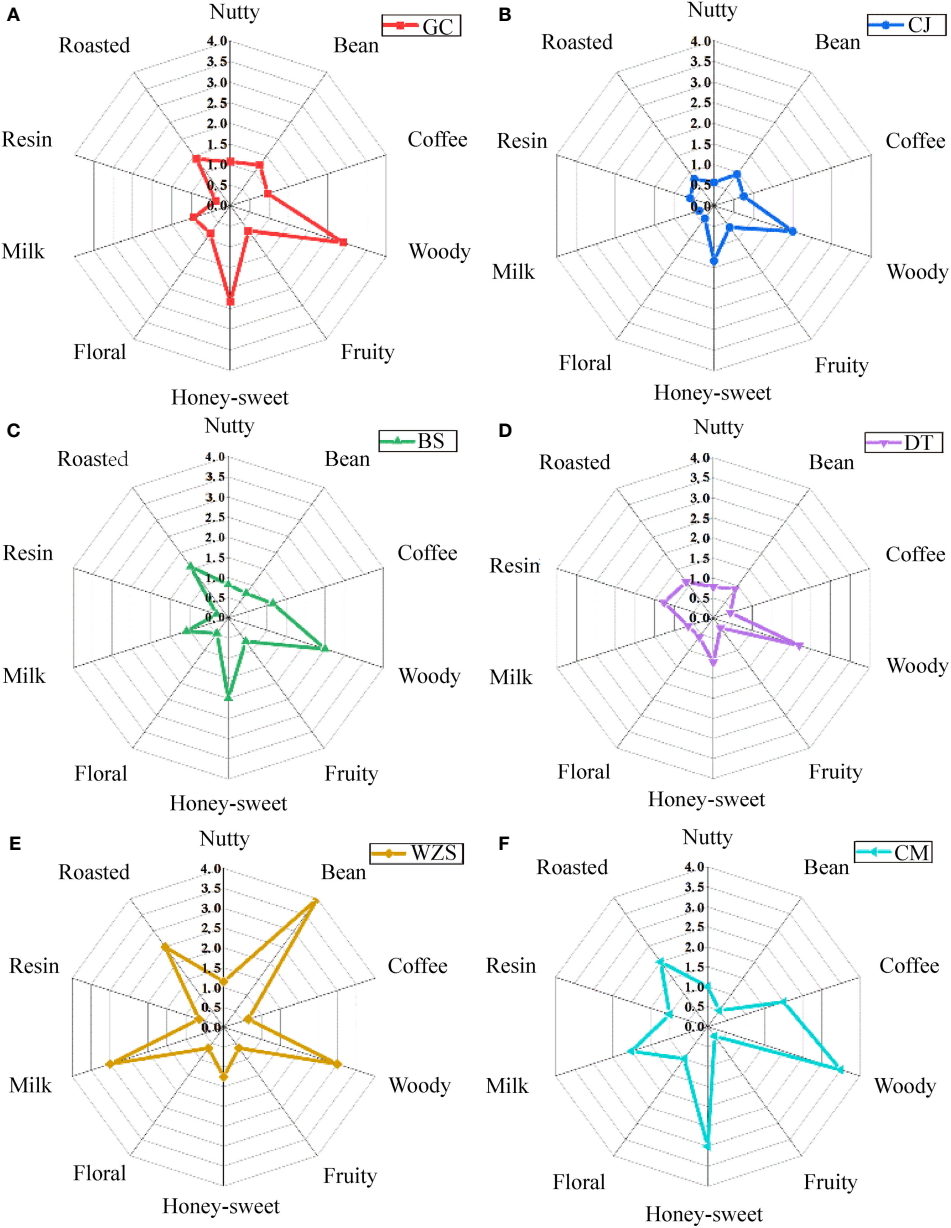
The flavor characteristics of cigar tobaccos from different origins of middle leaves. **(A)** Guangcun. **(B)** Changjiang. **(C)** Baisha. **(D)** Datian. **(E)** Wuzhishan. **(F)** Chengmai.

As shown in **Figure 5**, woody aroma was prominent in Guangcun, Changjiang, Baisha, Datian, and Chengmai, and bean aroma was prominent in Wuzhishan. Woody and honey-sweet aromas were dominant in Guangcun, supplemented by roasted, bean, fruit, and coffee aromas, while bean aroma was dominant in Wuzhishan, supplemented by woody, milk, and roasted aromas. The overall flavor characteristics of Guangcun and Changjiang were closer, while there were significant differences with all other origins. Different origins of flue-cured tobacco can be categorized according to the sensory quality of tobacco. Flue-cured tobacco in the main production areas of Henan was divided into three categories, and the total scores of the sensory quality were Jiaxian> Xiangcheng> Lushi> Lingbao> Linying> Fangcheng (Yan et al., 2013). The chemical composition and sensory quality of flue-cured tobacco have strong regional characteristics, and their characteristics from different origins with similar distances have high similarity (Hou et al., 2014).

**Figure 6:**
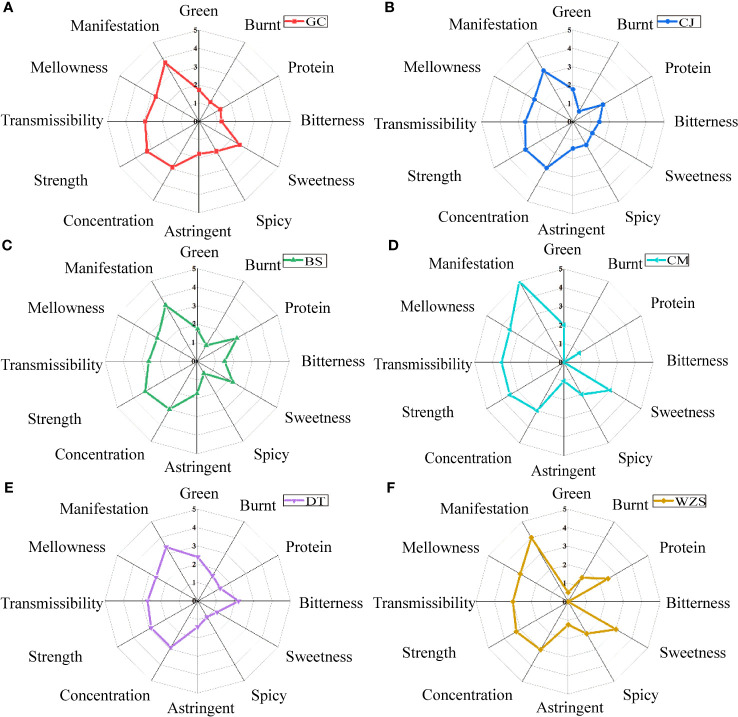
The style characteristics of cigar tobaccos from different origins of middle leaves. **(A)** Guangcun. **(B)** Changjiang. **(C)** Baisha. **(D)** Chengmai. **(E)** Datian. **(F)** Wuzhishan. The symbol * represented P<0.05.

The differences in style characteristics were shown in **Figure 6**, protein aroma was dominant in Baisha and Wuzhishan, and green aroma was dominant in Datian and Chengmai, probably due to the obvious green in Datian and Chengmai tobacco caused by immature harvest at the harvest stage or improperly during the curing process, and the elimination of green and the production of fragrance aroma as chlorophyll and chlorophyll alcohol were converted into neophytadiene in large amounts during the maturation of tobacco (You, 2015). Guangcun and Baisha were dominated by bitterness, Datian by sweetness, and Wuzhishan and Chengmai by sweetness and spiciness. The cigar-style manifestation of Wuzhishan and Chengmai was more significant. The sweetness in the eating flavor of cigar tobacco was prominent, probably due to the sweet aroma substances such as ketones and heterocyclic substances produced in the tobacco after combustion and smoking. Xiong et al. (2021) analyzed the water-soluble components of smoke particle phase matter of single-feed tobacco during combustion, and found that pyranones, furanones, furans, and cyclopentenones were the important sources of the flow fraction sweetness components by combining manual sensory taste and electronic tongue analysis.

**Figure 7:**
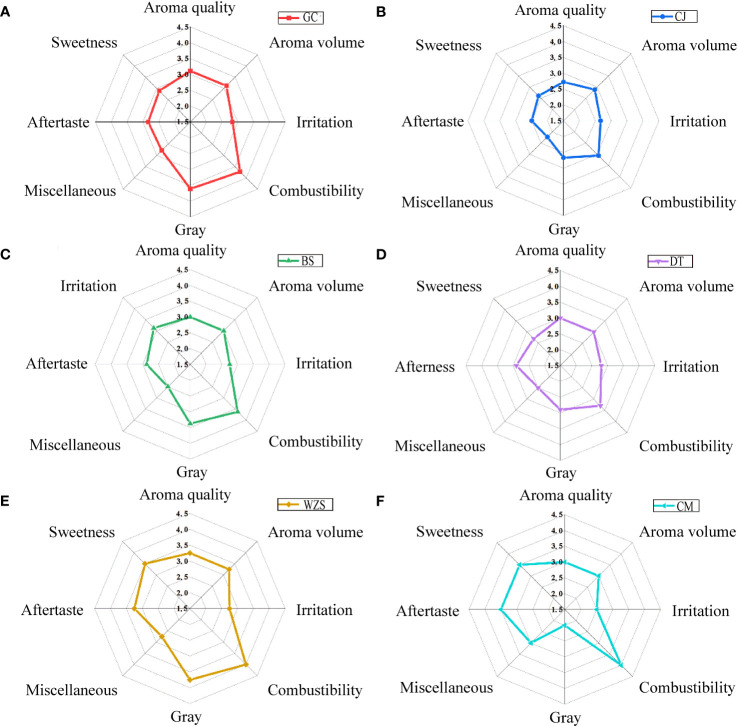
The quality characteristics of cigar tobaccos from different origins of middle leaves. **(A)** Guangcun. **(B)** Changjiang. **(C)** Baisha. **(D)** Datian. **(E)** Wuzhishan. **(F)** Chengmai.

The differences in quality characteristics were shown in **Figure 7**, the scores of Wuzhishan and Chengmai were higher, and Changjiang was lower. Among them, Wuzhishan had the highest scores for aroma quality, aroma volume, and gray, and Chengmai had the highest scores for miscellaneous aroma and aftertaste, but the lowest scores for irritation and gray, and both Wuzhishan and Chengmai had better combustibility. Changjiang had the lowest scores for aroma quality, aroma volume, combustibility, miscellaneous aroma, and aftertaste.

### 3.4 Correlation of aroma precursors with sensory characteristics

**Figure 8:**
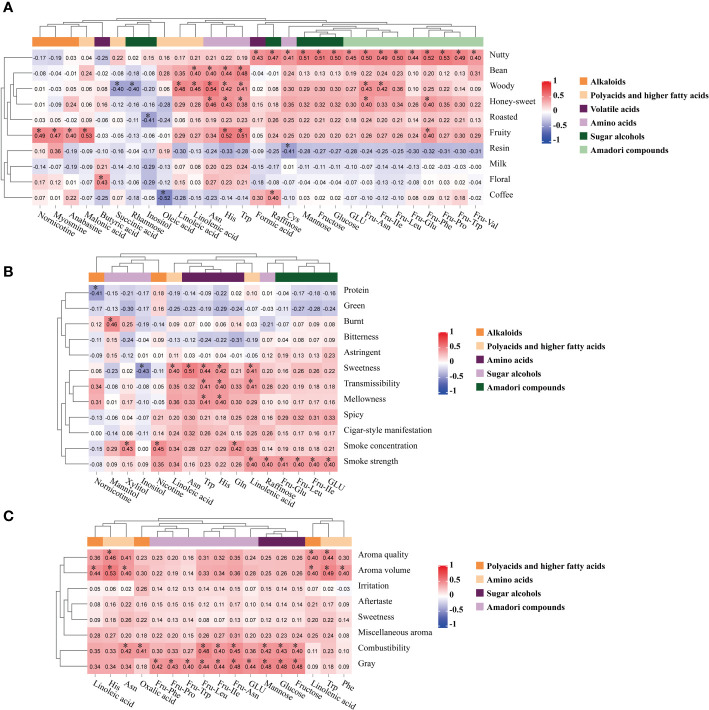
Pearson correlation between aroma precursors and sensory characteristics of cigar tobaccos from different origins of middle leaves, and * represented *P*<0.05. **(A)** Flavor. **(B)** Style. **(C)** Quality.

As shown in **Figure 8A**, and **Table S16**, aroma precursors were significantly correlated with flavor characteristics such as bean, woody, fruity, honey-sweet and nutty aromas, and aroma precursors with high correlations included alkaloids (nornicotine, myosmine, anabasine), organic acids (formic acid, butyric acid, malonic acid, succinic acid, oleic acid, linoleic acid, linolenic acid), amino acids (Asn, Trp, His, Cys), sugar alcohols (mannose, fructose, glucose, raffinose, inositol, rhamnose), and Amadori compounds (Fru-Asn, Fru-Glu, Fru-Leu, Fru-IIe, Fru-Phe, Fru-Pro, Fru-Trp, Fru-Val, GLU), polyphenols (neochlorogenic acid, chlorogenic acid, cryptochlorogenic acid, scopoletin, rutin). Within a certain range, the sensory scores of woody and nutty aromas showed an increasing trend with increasing contents of mannose, rhamnose, and nornicotine, and a decreasing trend with increasing contents of fructose, Ile, and glucose.

The aroma precursors were significantly correlated with the style characteristics of sweetness, smoke concentration, smoke strength, transmissibility, and mellowness (**Figure 8B** and **Table S17**), and the precursors with high correlations included alkaloids (nicotine, nornicotine), organic acids (linoleic acid, linolenic acid), amino acids (Asn, Trp, His, Gln), sugar alcohols (mannitol, xylitol, raffinose, inositol), Amadori compounds (Fru-Glu, Fru-Leu, Fru-IIe, GLU), polyphenols (neochlorogenic acid, chlorogenic acid, cryptochlorogenic acid, scopoletin, rutin). Among them, nornicotine was related to undesirable protein aroma, and there was also a negative correlation between nitrogenous compounds and sensory scores in flue-cured tobacco (Xia et al., 2015). Nicotine was significantly related to smoke concentration, which was the same as flue-cured tobacco (Chen et al., 2014). Inositol was closely related to sweetness, but other sugar alcohols such as raffinose, xylitol, and mannitol were not related. Fructose, glucose, sucrose, inositol, etc. were the main sources of the sweetness effects of tobacco leaf extracts (Zhang et al., 2016). The flavor and taste of tobacco were affected by the amino acids and the effect was related to the concentration. Asn, Trp, and His were all related to sweetness, indicating that their contents were in a reasonable range. Fru-Leu, Fru-IIe, GLU, and Fru-Glu did not emit aromas themselves, but their reaction after heating contributes to the smoke strength.

As shown in **Figure 8C** and **Table S18**, aroma precursors were significantly correlated with quality characteristics such as aroma quality, aroma volume, combustibility, and gray, and precursors with high correlations included organic acids (linoleic acid, linolenic acid, oxalic acid), amino acids (Asn, Trp, His, Phe), sugar alcohols (mannose, fructose, glucose), Amadori compounds (Fru-Asn, Fru-Leu, Fru-IIe, Fru-Phe, Fru-Pro, Fru-Trp, GLU), polyphenols (neochlorogenic acid, chlorogenic acid, cryptochlorogenic acid, scopoletin, rutin). There were 34 aroma precursors significantly related to the flavor characteristics, which were significantly more than the style and quality characteristics, indicating that the flavor characteristics were closely related to the aroma precursors. Linoleic acid and linolenic acid were related to aroma volume, due to the fat or spicy flavor of the tobacco was improved. Asn, His, Phe, and Trp improved the aroma quality and aroma volume in the current concentration, without causing irritation and bitterness. The intermediates of the Maillard reaction between amino acids and reducing sugars, Fru-Leu, Fru-IIe, Fru-Asn, and sugar alcohols included mannose, fructose, and glucose were all significantly correlated with combustibility and gray, while the difference in combustibility with flue-cured tobacco was smaller (Yan et al., 2013). Neochlorogenic acid, chlorogenic acid, cryptochlorogenic acid, scopoletin, and rutin were related to sweetness and miscellaneous aroma, while neochlorogenic acid, cryptochlorogenic acid, and scopoletin in cigars were closely correlated with aroma quality, aroma volume, and irritation (Xu et al., 2019). Besides, there were significant positive correlations between nicotine and aroma volume, concentration, irritation in flue-cured tobacco, significant negative correlations between anabasine, anatabine, and aftertaste, and significant typical correlation of anabasine, anatabine with aroma volume, concentration (Gao et al., 2008). The correlation between aroma precursors and sensory characteristics of cigar tobacco in the present study was significantly different from that of flue-cured tobacco, which may be related to tobacco varieties, processes, and the range of aroma precursors selected for analysis.

Based on the differential analysis of aroma precursors in different tobacco parts and origins, and the correlation analysis between the aroma precursors and sensory evaluation, the sensory characteristics could be regulated by changing the contents of nicotine, oxalic acid, Gln, Cys, and Fru-Pro in different tobacco parts, as well as the contents of myosmine, anabasine, mannitol, mannose, glucose, Gln, Trp, His, and Fru-Asn in different origins. The ecological environment and chemical composition of different producing areas varied greatly. Clarification of the relationship between the chemical composition and sensory quality from each producing area promoted the development of local high-quality tobacco leaves.

## 4 Conclusion

The compositions of the seven categories of aroma precursors were not changed by the tobacco parts and origins, but the contents were significantly affected. Cigar tobacco has a high conversion rate of nicotine, malic acid, oxalic acid, and citric acid were the dominant polyacids, acetic acid and formic acid were the dominant volatile acids, inositol, glucose, and fructose were high in sugar alcohols, Asp and GLU accounted for 67% and 58% of amino acids and Amadori compounds, respectively, and rutin and chlorogenic acid accounted for 85% of polyphenols. Tobacco parts mainly affected the amino acid content and slightly influenced the contents of polyacids and high fatty acids, volatile acids, alkaloids, and Amadori compounds, the contents of nicotine, total alkaloids, oxalic acid, total volatile acids, isovaleric acid, and neutral amino acids Cys, Gln, Gly, and Ile were significantly increased in upper leaves compared to middle leaves, while malic acid, acidic amino acids Glu and Asp, and Amadori compound Fru-Pro were decreased. The effect of tobacco origins over parts was more significant, mainly affecting the contents of amino acids, polyacids and high fatty acids, and sugar alcohols, and slightly influencing the contents of alkaloids, Amadori compounds, and volatile acids. Among them, the contents of myosmine, anabasine, total volatile acid, nonanoic acid, propanetriol, mannitol, mannose, glucose, Ala, Arg, Gln, Glu, His, Ser, Thr, Trp, Fru-Ala, and Fru-Asn were significantly affected. The flavor characteristics were prominent by wood aroma, and the style and quality characteristics varied greatly among different origins of middle leaves. There were 34, 21, and 22 aroma precursors with high correlations with flavor, style, and quality characteristics. The correlation analysis showed that the sensory characteristics could be regulated by changing the contents of nicotine, oxalic acid, Gln, Cys, and Fru-Pro in different tobacco parts, as well as the contents of myosmine, anabasine, mannitol, mannose, glucose, Gln, Trp, His, and Fru-Asn in different origins. It provided a basis to regulate the aroma precursors in different tobacco parts and origins to improve the sensory quality and industrial availability.

## Data availability statement

The original contributions presented in the study are included in the article/**Supplementary Material**. Further inquiries can be directed to the corresponding authors.

## Author contributions

ZG: Formal Analysis, Investigation, Software, Validation, Writing – original draft. PH: Data curation, Software, Visualization, Validation, Writing – original draft. HG: Resources, Supervision, Investigation, Writing – review & editing. JL: Conceptualization, Methodology, Supervision, Writing – review & editing. JQ: Conceptualization, Funding acquisition, Project administration, Writing – review & editing. BC: Data curation, Methodology, Software, Writing – original draft.
