# Supplementary material for: Aroma precursors of cigars from different tobacco parts and origins, and their correlations with sensory characteristics
Source: Front Plant Sci. 2023 Dec 13;14:1264739. doi: 10.3389/fpls.2023.1264739 (PMC10773810; doi:10.3389/fpls.2023.1264739)
Supplement: Supplementary file 1 [file Table_1.docx]

**Table S1** Information of cigar samples.

| No. | Origins | Years | Varieties | Parts | No. | Origins | Years | Varieties | Parts |
| --- | --- | --- | --- | --- | --- | --- | --- | --- | --- |
| 1 | Guangcun | 2017 | Hainan2 | Middle | 32 | Changjiang | 2020 | Guyin4 | Upper |
| 2 | Guangcun | 2017 | Hainan2 | Upper | 33 | Changjiang | 2021 | Hainan2 | Middle |
| 3 | Guangcun | 2018 | Hainan2 | Middle | 34 | Changjiang | 2021 | Hainan2 | Upper |
| 4 | Guangcun | 2018 | Hainan2 | Upper | 35 | Changjiang | 2021 | Hainan3 | Middle |
| 5 | Guangcun | 2019 | Hainan2 | Middle | 36 | Changjiang | 2021 | Hainan3 | Upper |
| 6 | Guangcun | 2019 | Hainan2 | Upper | 37 | Baisha | 2017 | Hainan2 | Middle |
| 7 | Guangcun | 2019 | Hainan3 | Middle | 38 | Baisha | 2017 | Hainan2 | Upper |
| 8 | Guangcun | 2019 | Hainan3 | Upper | 39 | Baisha | 2018 | Hainan2 | Middle |
| 9 | Guangcun | 2020 | Hainan2 | Middle | 40 | Baisha | 2018 | Hainan2 | Upper |
| 10 | Guangcun | 2020 | Hainan2 | Upper | 41 | Baisha | 2020 | Hainan2 | Middle |
| 11 | Guangcun | 2020 | Hainan3 | Middle | 42 | Baisha | 2020 | Hainan2 | Upper |
| 12 | Guangcun | 2020 | Hainan3 | Upper | 43 | Baisha | 2021 | Hainan3 | Middle |
| 13 | Guangcun | 2021 | Hainan2 | Middle | 44 | Baisha | 2021 | Hainan3 | Upper |
| 14 | Guangcun | 2021 | Hainan2 | Upper | 45 | Datian | 2020 | YS01 | Upper |
| 15 | Guangcun | 2021 | Hainan3 | Middle | 46 | Datian | 2020 | YS01 | Middle |
| 16 | Guangcun | 2021 | Hainan3 | Upper | 47 | Wanzhong | 2020 | YS01 | Upper |
| 17 | Guangcun | 2021 | Guyin4 | Middle | 48 | Datian | 2020 | YS02 | Upper |
| 18 | Guangcun | 2021 | Guyin4 | Upper | 49 | Datian | 2020 | YS02 | Middle |
| 19 | Changjiang | 2017 | Hainan2 | Middle | 50 | Datian | 2021 | YS01 | Upper |
| 20 | Changjiang | 2017 | Hainan2 | Upper | 51 | Datian | 2021 | YS01 | Middle |
| 21 | Changjiang | 2018 | Hainan2 | Middle | 52 | Datian | 2021 | YS02 | Upper |
| 22 | Changjiang | 2018 | Hainan2 | Upper | 53 | Datian | 2021 | YS02 | Middle |
| 23 | Changjiang | 2019 | Jianheng1 | Middle | 54 | Datian | 2021 | YS03 | Upper |
| 24 | Changjiang | 2019 | Jianheng1 | Upper | 55 | Datian | 2021 | YS03 | Middle |
| 25 | Changjiang | 2019 | Hainan3 | Middle | 56 | Wuzhishan | 2021 | T4/T5 | Upper |
| 26 | Changjiang | 2019 | Hainan3 | Upper | 57 | Wuzhishan | 2021 | T4/T5 | Middle |
| 27 | Changjiang | 2020 | Hainan2 | Middle | 58 | Wuzhishan | 2021 | T11/T12 | Upper |
| 28 | Changjiang | 2020 | Hainan2 | Upper | 59 | Wuzhishan | 2021 | T11/T12 | Middle |
| 29 | Changjiang | 2020 | Jianheng3 | Middle | 60 | Chengmai | 2021 | 166A | Upper |
| 30 | Changjiang | 2020 | Jianheng3 | Upper | 61 | Chengmai | 2021 | 166A | Middle |
| 31 | Changjiang | 2020 | Guyin4 | Middle |  |  |  |  |  |

**Table S2** The alkaloids contents in cigar tobaccos of different parts (mg/g).

| Indices | Parts | Numbers | Concentration (mg/g) | Maximum | Minimum | Variation  coefficient |
| --- | --- | --- | --- | --- | --- | --- |
| Nicotine | Upper | 31 | 33.97±11.53^a^ | 54.96 | 8.14 | 4.51 |
|  | Middle | 30 | 28.52±6.91^b^ | 43.80 | 11.2 | 24.25 |
|  | Total | 61 | 31.29±9.86 | 54.96 | 8.14 | 31.60 |
| Nornicotine | Upper | 31 | 2.07±2.13^a^ | 9.57 | 0.31 | 102.90 |
|  | Middle | 30 | 1.66±1.40^a^ | 5.92 | 0.34 | 84.34 |
|  | Total | 61 | 1.87±1.81 | 9.57 | 0.31 | 96.79 |
| Myosmine | Upper | 31 | 0.07±0.05^a^ | 0.22 | 0.04 | 71.43 |
|  | Middle | 30 | 0.07±0.05^a^ | 0.23 | 0.03 | 71.43 |
|  | Total | 61 | 0.07±0.05 | 0.23 | 0.03 | 71.43 |
| Anabasine | Upper | 31 | 0.20±0.07^a^ | 0.36 | 0.12 | 35.00 |
|  | Middle | 30 | 0.16±0.04^a^ | 0.25 | 0.11 | 25.00 |
|  | Total | 61 | 0.18±0.06 | 0.36 | 0.11 | 33.33 |
| Anatabine | Upper | 31 | 1.44±0.94^a^ | 3.20 | 0.19 | 65.28 |
|  | Middle | 30 | 1.21±0.55^a^ | 2.22 | 0.30 | 45.45 |
|  | Total | 61 | 1.33±0.78 | 3.20 | 0.19 | 58.65 |
| Total | Upper | 31 | 37.75±11.83^a^ | 61.03 | 14.89 | 4.85 |
|  | Middle | 30 | 31.61±6.60^b^ | 48.40 | 18.39 | 20.89 |
|  | Total | 61 | 34.73±10.03 | 61.03 | 14.89 | 0.09 |
| Nicotine  conversion rate (%) | Upper | 31 | 6.91±10.99^a^ | 54.04 | 0.95 | 159.04 |
|  | Middle | 30 | 6.25±7.41^a^ | 31.67 | 1.07 | 118.56 |
|  | Total | 61 | 6.59±9.33 | 54.04 | 0.95 | 141.58 |

Data are the means ± standard deviation, and the total represents a mixture of the upper and middle leaves

Values in the same column of a compound with different letters indicate significant difference (*P*<0.05) according to Duncan’s tests

**Table S3** The polyacids and higher fatty acids contents in cigar tobaccos of different parts (mg/g).

| Indices | Parts | Numbers | Concentration  (mg/g) | Maximum | Minimum | Variation  coefficient |
| --- | --- | --- | --- | --- | --- | --- |
| Oxalic acid | Upper | 31 | 29.84±5.07^a^ | 41.32 | 21.55 | 17.01 |
|  | Middle | 30 | 25.91±3.37^b^ | 31.9 | 17.99 | 13.01 |
|  | Total | 61 | 27.91±4.72 | 41.32 | 17.99 | 16.92 |
| Malonic acid | Upper | 31 | 1.87±0.48^a^ | 2.70 | 0.86 | 25.67 |
|  | Middle | 30 | 1.95±0.37^a^ | 2.82 | 1.12 | 18.97 |
|  | Total | 61 | 1.91±0.43 | 2.82 | 0.86 | 22.51 |
| Succinic acid | Upper | 31 | 1.07±1.38^a^ | 5.68 | 0.16 | 128.97 |
|  | Middle | 30 | 1.17±1.87^a^ | 7.75 | 0.23 | 159.83 |
|  | Total | 61 | 1.12±1.63 | 7.75 | 0.16 | 145.54 |
| Malic acid | Upper | 31 | 44.04±19.92^a^ | 74.67 | 10.03 | 22.55 |
|  | Middle | 30 | 47.29±15.44^b^ | 75.73 | 22.61 | 11.53 |
|  | Total | 61 | 45.64±17.79 | 75.73 | 10.03 | 17.08 |
| Palmitic acid | Upper | 31 | 0.52±0.15^a^ | 0.86 | 0.27 | 28.85 |
|  | Middle | 30 | 0.57±0.15^a^ | 0.86 | 0.31 | 26.32 |
|  | Total | 61 | 0.55±0.15 | 0.86 | 0.27 | 27.27 |
| Citric acid | Upper | 31 | 16.85±8.77^a^ | 53.35 | 5.05 | 52.20 |
|  | Middle | 30 | 18.50±9.63^a^ | 60.68 | 8.53 | 52.05 |
|  | Total | 61 | 17.66±9.17 | 60.68 | 5.05 | 52.10 |
| Oleic acid | Upper | 31 | 0.20±0.07^a^ | 0.36 | 0.12 | 35.00 |
|  | Middle | 30 | 0.19±0.05^a^ | 0.32 | 0.11 | 26.32 |
|  | Total | 61 | 0.20±0.06 | 0.36 | 0.11 | 30.00 |
| Linoleic acid | Upper | 31 | 0.36±0.16^a^ | 0.78 | 0.15 | 44.44 |
|  | Middle | 30 | 0.32±0.13^a^ | 0.70 | 0.14 | 40.63 |
|  | Total | 61 | 0.34±0.15 | 0.78 | 0.14 | 44.12 |
| Linolenic acid | Upper | 31 | 0.33±0.23^a^ | 1.14 | 0.06 | 69.70 |
|  | Middle | 30 | 0.31±0.18^a^ | 0.91 | 0.12 | 58.06 |
|  | Total | 61 | 0.32±0.20 | 1.14 | 0.06 | 62.50 |
| Total | Upper | 31 | 95.08±23.43^a^ | 147.10 | 51.24 | 3.61 |
|  | Middle | 30 | 96.21±15.73^a^ | 122.08 | 70.84 | 5.96 |
|  | Total | 61 | 95.64±19.86 | 147.10 | 51.24 | 10.31 |

Data are the means ± standard deviation

Values in the same column of a compound with different letters indicate significant difference (*P*<0.05) according to Duncan’s tests

The total represents a mixture of upper and middle leaves

**Table S4** The volatile acids contents in cigar tobaccos of different parts (μg/g).

| Indices | Parts | Numbers | Concentration  (μg/g) | Maximum | Minimum | Variation  coefficient |
| --- | --- | --- | --- | --- | --- | --- |
| Acetic acid | Upper | 31 | 46.00±30.74^a^ | 184.79 | 18.38 | 1.61 |
|  | Middle | 30 | 45.88±46.35^a^ | 271.75 | 13.20 | 13.86 |
|  | Total | 61 | 45.94±38.87 | 271.75 | 13.20 | 19.32 |
| Formic acid | Upper | 31 | 89.25±5.46^a^ | 112.05 | 84.84 | 6.12 |
|  | Middle | 30 | 89.28±6.92^a^ | 122.73 | 84.85 | 7.76 |
|  | Total | 61 | 89.26±6.17 | 122.73 | 84.84 | 6.92 |
| Propionic acid | Upper | 31 | 1.16±0.83^a^ | 4.53 | 0.001 | 71.55 |
|  | Middle | 30 | 1.19±0.88^a^ | 3.81 | 0.001 | 73.95 |
|  | Total | 61 | 1.17±0.85 | 4.53 | 0.001 | 72.65 |
| Isobutyric acid | Upper | 31 | 2.68±2.26^a^ | 12.51 | 1.03 | 84.33 |
|  | Middle | 30 | 2.06±1.66^a^ | 7.66 | 0.53 | 80.58 |
|  | Total | 61 | 2.38±1.99 | 12.51 | 0.53 | 83.61 |
| Butyric acid | Upper | 31 | 0.96±0.69^a^ | 3.80 | 0.30 | 71.88 |
|  | Middle | 30 | 0.86±0.83^a^ | 4.75 | 0.27 | 96.51 |
|  | Total | 61 | 0.91±0.76 | 4.75 | 0.27 | 83.52 |
| Isovaleric acid | Upper | 31 | 26.55±16.16^a^ | 94.51 | 9.80 | 23.25 |
|  | Middle | 30 | 21.19±13.57^b^ | 65.67 | 5.16 | 16.92 |
|  | Total | 61 | 23.91±15.06 | 94.51 | 5.16 | 21.17 |
| Valeric acid | Upper | 31 | 0.74±0.30^a^ | 1.63 | 0.34 | 40.54 |
|  | Middle | 30 | 0.70±0.37^a^ | 2.09 | 0.21 | 52.86 |
|  | Total | 61 | 0.72±0.33 | 2.09 | 0.21 | 45.83 |
| Hexanoic acid | Upper | 31 | 2.91±3.89^a^ | 11.46 | 0.11 | 133.68 |
|  | Middle | 30 | 1.30±2.63^a^ | 9.61 | 0.11 | 202.31 |
|  | Total | 61 | 2.12±3.40 | 11.46 | 0.11 | 160.38 |
| Octanoic acid | Upper | 31 | 1.10±0.79^a^ | 3.16 | 0.18 | 71.82 |
|  | Middle | 30 | 1.04±0.89^a^ | 3.88 | 0.00 | 85.58 |
|  | Total | 61 | 1.07±0.83 | 3.88 | 0.00 | 77.57 |
| Nonanoic acid | Upper | 31 | 1.11±0.57^a^ | 2.20 | 0.40 | 51.35 |
|  | Middle | 30 | 1.42±1.47^a^ | 8.33 | 0.38 | 103.52 |
|  | Total | 61 | 1.26±1.11 | 8.33 | 0.38 | 88.10 |
| Total | Upper | 31 | 172.45±46.14^a^ | 325.24 | 127.00 | 3.57 |
|  | Middle | 30 | 164.93±59.54^b^ | 425.38 | 113.01 | 5.82 |
|  | Total | 61 | 168.75±52.84 | 425.38 | 113.01 | 1.69 |

Data are the means ± standard deviation

Values in the same column of a compound with different letters indicate significant difference (*P*<0.05) according to Duncan’s tests

The total represents a mixture of upper and middle leaves

**Table S5** The sugar alcohols contents in cigar tobaccos of different parts (mg/g).

| Indices | Parts | Numbers | Concentration  (mg/g) | Maximum | Minimum | Variation  coefficient |
| --- | --- | --- | --- | --- | --- | --- |
| Propanetriol | Upper | 31 | 0.36±0.27^a^ | 1.38 | 0.04 | 75.00 |
|  | Middle | 30 | 0.43±0.34^a^ | 1.61 | 0.08 | 79.07 |
|  | Total | 61 | 0.39±0.31 | 1.61 | 0.04 | 79.49 |
| Mannitol | Upper | 31 | 0.20±0.17^a^ | 0.87 | 0.03 | 85.00 |
|  | Middle | 30 | 0.16±0.06^a^ | 0.32 | 0.05 | 37.50 |
|  | Total | 61 | 0.18±0.13 | 0.87 | 0.03 | 72.22 |
| Mannose | Upper | 31 | 0.31±0.32^a^ | 1.53 | 0.07 | 103.23 |
|  | Middle | 30 | 0.42±0.70^a^ | 3.33 | 0.06 | 166.67 |
|  | Total | 61 | 0.37±0.54 | 3.33 | 0.06 | 145.95 |
| Fructose | Upper | 31 | 0.44±0.47^a^ | 2.19 | 0.06 | 106.82 |
|  | Middle | 30 | 0.60±1.07^a^ | 5.15 | 0.07 | 178.33 |
|  | Total | 61 | 0.52±0.82 | 5.15 | 0.06 | 157.69 |
| Inositol | Upper | 31 | 1.11±0.87^a^ | 4.22 | 0.14 | 78.38 |
|  | Middle | 30 | 1.10±0.76^a^ | 3.24 | 0.22 | 69.09 |
|  | Total | 61 | 1.10±0.81 | 4.22 | 0.14 | 73.64 |
| Xylitol | Upper | 31 | 0.13±0.08^a^ | 0.34 | 0.02 | 61.54 |
|  | Middle | 30 | 0.12±0.05^a^ | 0.21 | 0.04 | 41.67 |
|  | Total | 61 | 0.13±0.06 | 0.34 | 0.02 | 46.15 |
| Raffinose  （μg/g） | Upper | 31 | 8.86±7.06^a^ | 40.65 | 4.02 | 79.68 |
|  | Middle | 30 | 9.06±7.00^a^ | 42.09 | 4.82 | 77.26 |
|  | Total | 61 | 8.96±6.98 | 42.09 | 4.02 | 77.90 |
| Glucose | Upper | 31 | 0.48±0.51^a^ | 2.38 | 0.09 | 106.25 |
|  | Middle | 30 | 0.68±1.19^a^ | 5.74 | 0.08 | 175.00 |
|  | Total | 61 | 0.58±0.91 | 5.74 | 0.08 | 156.90 |
| Rhamnose  （μg/g） | Upper | 31 | 58.66±33.29^a^ | 163.16 | 6.40 | 5.61 |
|  | Middle | 30 | 37.69±21.16^a^ | 107.08 | 4.47 | 3.09 |
|  | Total | 61 | 48.35±29.70 | 163.16 | 4.47 | 61.49 |
| Sucrose | Upper | 31 | 0.11±0.13^a^ | 0.67 | 0.02 | 118.18 |
|  | Middle | 30 | 0.13±0.21^a^ | 1.17 | 0.02 | 161.54 |
|  | Total | 61 | 0.12±0.17 | 1.17 | 0.02 | 141.67 |
| Total | Upper | 31 | 3.20±2.00^a^ | 8.95 | 0.89 | 62.50 |
|  | Middle | 30 | 3.69±3.45^a^ | 17.10 | 1.17 | 93.50 |
|  | Total | 61 | 3.44±2.80 | 17.10 | 0.89 | 81.40 |

Data are the means ± standard deviation

Values in the same column of a compound with different letters indicate significant difference (*P*<0.05) according to Duncan’s tests

The total represents a mixture of upper and middle leaves

**Table S6** The amino acids contents in cigar tobaccos of different parts (mg/g).

| Indices | Parts | Numbers | Concentration  (mg/g) | Maximum | Minimum | Variation  coefficient |
| --- | --- | --- | --- | --- | --- | --- |
| Ala | Upper | 31 | 0.31±0.25^a^ | 0.74 | 0.02 | 78.24 |
|  | Middle | 30 | 0.25±0.15^a^ | 0.65 | 0.04 | 59.18 |
|  | Total | 61 | 0.28±0.20 | 0.74 | 0.02 | 72.37 |
| Arg | Upper | 31 | 0.05±0.03^a^ | 0.12 | 0.01 | 60.31 |
|  | Middle | 30 | 0.04±0.02^a^ | 0.09 | 0.01 | 44.53 |
|  | Total | 61 | 0.05±0.02 | 0.12 | 0.01 | 53.27 |
| Asn | Upper | 31 | 0.81±1.24^a^ | 4.80 | 0.02 | 153.10 |
|  | Middle | 30 | 0.73±0.86^a^ | 2.90 | 0.02 | 118.14 |
|  | Total | 61 | 0.77±1.06 | 4.80 | 0.02 | 137.79 |
| Asp | Upper | 31 | 8.14±3.77^a^ | 14.60 | 1.50 | 46.26 |
|  | Middle | 30 | 9.81±3.30^b^ | 17.13 | 4.14 | 33.69 |
|  | Total | 61 | 8.96±3.62 | 17.13 | 1.50 | 40.36 |
| Cys  (μg/g) | Upper | 31 | 5.64±0.08^a^ | 5.88 | 5.55 | 1.42 |
|  | Middle | 30 | 5.67±0.10^b^ | 6.06 | 5.55 | 1.76 |
|  | Total | 61 | 5.65±0.09 | 6.06 | 5.55 | 1.59 |
| Gln | Upper | 31 | 0.21±0.14^a^ | 0.49 | 0.02 | 68.82 |
|  | Middle | 30 | 0.19±0.09^b^ | 0.34 | 0.04 | 47.98 |
|  | Total | 61 | 0.20±0.12 | 0.49 | 0.02 | 60.07 |
| Glu | Upper | 31 | 0.77±0.52^a^ | 1.76 | 0.08 | 66.94 |
|  | Middle | 30 | 0.85±0.45^b^ | 2.26 | 0.22 | 52.40 |
|  | Total | 61 | 0.81±0.48 | 2.26 | 0.08 | 59.30 |
| Gly | Upper | 31 | 0.10±0.06^a^ | 0.23 | 0.01 | 57.01 |
|  | Middle | 30 | 0.09±0.05^b^ | 0.19 | 0.02 | 54.93 |
|  | Total | 61 | 0.10±0.06 | 0.23 | 0.01 | 5.49 |
| His | Upper | 31 | 0.20±0.10^a^ | 0.48 | 0.08 | 3.98 |
|  | Middle | 30 | 0.19±0.08^a^ | 0.43 | 0.09 | 0.54 |
|  | Total | 61 | 0.20±0.09 | 0.48 | 0.08 | 4.82 |
| Ile | Upper | 31 | 0.06±0.05^a^ | 0.17 | 0.01 | 8.64 |
|  | Middle | 30 | 0.05±0.03^b^ | 0.12 | 0.01 | 17.78 |
|  | Total | 61 | 0.06±0.04 | 0.17 | 0.01 | 14.64 |
| Leu | Upper | 31 | 0.11±0.09^a^ | 0.34 | 0.01 | 6.26 |
|  | Middle | 30 | 0.10±0.05^a^ | 0.21 | 0.01 | 4.87 |
|  | Total | 61 | 0.11±0.07 | 0.34 | 0.01 | 2.80 |
| lys | Upper | 31 | 0.10±0.07^a^ | 0.26 | 0.01 | 2.15 |
|  | Middle | 30 | 0.09±0.05^a^ | 0.20 | 0.02 | 1.96 |
|  | Total | 61 | 0.10±0.06 | 0.26 | 0.01 | 2.56 |
| Phe | Upper | 31 | 0.22±0.21^a^ | 0.68 | 0.01 | 96.78 |
|  | Middle | 30 | 0.19±0.16^a^ | 0.56 | 0.01 | 83.79 |
|  | Total | 61 | 0.21±0.19 | 0.68 | 0.01 | 91.40 |
| Pro | Upper | 31 | 0.93±0.81^a^ | 2.68 | 0.04 | 86.49 |
|  | Middle | 30 | 0.83±0.57^a^ | 1.92 | 0.12 | 68.69 |
|  | Total | 61 | 0.88±0.70 | 2.68 | 0.04 | 79.04 |
| Ser | Upper | 31 | 0.19±0.16^a^ | 0.63 | 0.01 | 87.84 |
|  | Middle | 30 | 0.21±0.14^a^ | 0.51 | 0.02 | 68.82 |
|  | Total | 61 | 0.20±0.15 | 0.63 | 0.01 | 77.68 |
| Thr | Upper | 31 | 0.42±0.19^a^ | 0.89 | 0.12 | 46.02 |
|  | Middle | 30 | 0.41±0.15^a^ | 0.74 | 0.200 | 36.88 |
|  | Total | 61 | 0.42±0.17 | 0.89 | 0.12 | 41.49 |
| Trp | Upper | 31 | 0.07±0.08^a^ | 0.36 | 0.01 | 111.79 |
|  | Middle | 30 | 0.07±0.06^a^ | 0.28 | 0.01 | 91.86 |
|  | Total | 61 | 0.07±0.07 | 0.36 | 0.01 | 101.77 |
| Tyr | Upper | 31 | 0.05±0.04^a^ | 0.12 | 0.00 | 84.29 |
|  | Middle | 30 | 0.04±0.03^a^ | 0.12 | 0.01 | 73.62 |
|  | Total | 61 | 0.05±0.04 | 0.12 | 0.00 | 79.52 |
| Val | Upper | 31 | 0.19±0.16^a^ | 0.52 | 0.01 | 83.98 |
|  | Middle | 30 | 0.17±0.12^a^ | 0.47 | 0.03 | 68.96 |
|  | Total | 61 | 0.18±0.14 | 0.52 | 0.01 | 77.14 |
| Total | Upper | 31 | 12.70±6.27^a^ | 21.75 | 2.18 | 49.25 |
|  | Middle | 30 | 14.12±4.88^a^ | 23.88 | 5.69 | 34.56 |
|  | Total | 61 | 13.41±5.62 | 23.88 | 2.18 | 41.91 |

Data are the means ± standard deviation

Values in the same column of a compound with different letters indicate significant difference (*P*<0.05) according to Duncan’s tests

The total represents a mixture of upper and middle leaves

**Table S7** The Amadori compounds contents in cigar tobaccos of different parts (μg/g).

| Indices | Parts | Numbers | Concentration  (μg/g) | Maximum | Minimum | Variation  coefficient |
| --- | --- | --- | --- | --- | --- | --- |
| Fru-Ala | Upper | 31 | 46.37±24.38^a^ | 113.34 | 13.53 | 9.45 |
|  | Middle | 30 | 42.79±26.92^a^ | 151.24 | 17.34 | 16.17 |
|  | Total | 61 | 44.61±25.51 | 151.24 | 13.53 | 12.35 |
| Fru-Asn | Upper | 31 | 31.51±59.92^a^ | 285.37 | 4.91 | 31.48 |
|  | Middle | 30 | 38.57±82.80^a^ | 429.45 | 5.69 | 214.67 |
|  | Total | 61 | 34.98±71.57 | 429.45 | 4.91 | 4.49 |
| Fru-Glu | Upper | 31 | 31.28±18.38^a^ | 79.77 | 10.66 | 26.79 |
|  | Middle | 30 | 36.05±30.79^a^ | 154.88 | 12.57 | 2.19 |
|  | Total | 61 | 33.63±25.16 | 154.88 | 10.66 | 15.34 |
| Fru-Gly | Upper | 31 | 34.08±33.72^a^ | 136.88 | 4.91 | 10.92 |
|  | Middle | 30 | 32.79±42.69^a^ | 227.12 | 6.36 | 8.20 |
|  | Total | 61 | 33.45±38.07 | 227.12 | 4.91 | 24.13 |
| Fru-Leu | Upper | 31 | 2.07±2.39^a^ | 10.54 | 0.16 | 115.46 |
|  | Middle | 30 | 1.74±2.73^a^ | 13.83 | 0.20 | 156.90 |
|  | Total | 61 | 1.91±2.54 | 13.83 | 0.16 | 132.98 |
| Fru-IIe | Upper | 31 | 2.15±2.46^a^ | 11.18 | 0.19 | 114.42 |
|  | Middle | 30 | 1.80±2.68^a^ | 13.94 | 0.17 | 148.89 |
|  | Total | 61 | 1.98±2.55 | 13.94 | 0.17 | 128.79 |
| Fru-Phe | Upper | 31 | 9.23±11.56^a^ | 57.44 | 1.90 | 16.90 |
|  | Middle | 30 | 8.44±10.43^a^ | 46.76 | 1.97 | 5.09 |
|  | Total | 61 | 8.84±10.93 | 57.44 | 1.90 | 10.52 |
| Fru-Pro | Upper | 31 | 87.39±78.89^a^ | 317.00 | 18.78 | 10.17 |
|  | Middle | 30 | 102.59±151.83^b^ | 718.15 | 17.15 | 1.79 |
|  | Total | 61 | 94.87±119.63 | 718.15 | 17.15 | 10.15 |
| Fru-Trp | Upper | 31 | 5.20±4.83^a^ | 24.04 | 1.30 | 92.88 |
|  | Middle | 30 | 5.63±7.70^a^ | 40.34 | 1.20 | 136.77 |
|  | Total | 61 | 5.41±6.36 | 40.34 | 1.20 | 117.56 |
| Fru-Val | Upper | 31 | 3.91±2.53^a^ | 11.04 | 0.47 | 64.71 |
|  | Middle | 30 | 3.50±2.42^a^ | 11.78 | 1.31 | 69.14 |
|  | Total | 61 | 3.71±2.47 | 11.78 | 0.47 | 66.58 |
| GLU | Upper | 31 | 370.02±339.25^a^ | 1474.47 | 31.77 | 2.50 |
|  | Middle | 30 | 360.32±417.91^a^ | 1926.41 | 54.95 | 2.20 |
|  | Total | 61 | 365.25±376.81 | 1926.41 | 31.77 | 1.86 |
| Total | Upper | 31 | 623.21±523.54^a^ | 2400.46 | 116.53 | 0.57 |
|  | Middle | 30 | 634.23±704.79^a^ | 3157.27 | 173.41 | 0.76 |
|  | Total | 61 | 628.63±614.13 | 3157.27 | 116.53 | 0.66 |

Data are the means ± standard deviation

Values in the same column of a compound with different letters indicate significant difference (*P*<0.05) according to Duncan’s tests

The total represents a mixture of upper and middle leaves

**Table S8** The polyphenols contents in cigar tobaccos of different parts (mg/g).

| Indices | Parts | Numbers | Concentration  (mg/g) | Maximum | Minimum | Variation  coefficient |
| --- | --- | --- | --- | --- | --- | --- |
| Neochlorogenic acid | Upper | 31 | 0.02±0.01^a^ | 0.04 | 0.01 | 50.00 |
|  | Middle | 30 | 0.02±0.01^a^ | 0.03 | 0.01 | 50.00 |
|  | Total | 61 | 0.02±0.01 | 0.04 | 0.01 | 50.00 |
| Chlorogenic acid | Upper | 31 | 0.48±0.25^a^ | 0.58 | 0.37 | 52.08 |
|  | Middle | 30 | 0.48±0.20^a^ | 0.58 | 0.37 | 41.67 |
|  | Total | 61 | 0.48±0.22 | 0.58 | 0.37 | 45.83 |
| Cryptochlorogenic acid | Upper | 31 | 0.13±0.06^a^ | 0.14 | 0.11 | 46.15 |
|  | Middle | 30 | 0.13±0.08^a^ | 0.17 | 0.11 | 61.54 |
|  | Total | 61 | 0.13±0.07 | 0.17 | 0.11 | 53.85 |
| Scopoletin | Upper | 31 | 0.03±0.02^a^ | 0.04 | 0.02 | 66.67 |
|  | Middle | 30 | 0.03±0.02^a^ | 0.05 | 0.02 | 66.67 |
|  | Total | 61 | 0.03±0.02 | 0.05 | 0.02 | 66.67 |
| Rutin | Upper | 31 | 0.50±0.41^a^ | 0.58 | 0.42 | 82.00 |
|  | Middle | 30 | 0.54±0.49^a^ | 0.59 | 0.48 | 90.74 |
|  | Total | 61 | 0.52±0.45 | 0.59 | 0.42 | 86.54 |
| Total | Upper | 31 | 1.15±0.66^a^ | 1.34 | 0.99 | 57.39 |
|  | Middle | 30 | 1.20±0.82^a^ | 1.26 | 1.10 | 68.33 |
|  | Total | 61 | 1.18±0.74 | 1.34 | 0.99 | 62.71 |

Data are the means ± standard deviation

Values in the same column of a compound with different letters indicate significant difference (*P*<0.05) according to Duncan’s tests

The total represents a mixture of upper and middle leaves

**Table S9** The alkaloids contents in cigar tobaccos of different origins (mg/g).

| Indices | Parts | Guangcun | Changjiang | Baisha | Datian | Wuzhishan | Chengmai |
| --- | --- | --- | --- | --- | --- | --- | --- |
| Nicotine | Upper | 41.71±12.02^a^ | 32.10±15.14^a^ | 27.66±3.77^a^ | 31.59±5.73^a^ | 29.46±1.03^a^ | 29.22^a^ |
|  | Middle | 27.62±8.74^a^ | 28.97±8.94^a^ | 29.85±1.35^a^ | 27.47±3.82^a^ | 27.3±1.84^a^ | 34.99^a^ |
|  | Total | 34.66±12.51^a^ | 30.53±12.17^a^ | 28.76±2.87^a^ | 29.53±5.08^a^ | 28.38±1.74^a^ | 32.11^a^ |
| Nornicotine | Upper | 3.18±2.07^a^ | 2.31±2.80^a^ | 1.08±1.28^a^ | 1.84±1.33^a^ | 0.34±0.04^a^ | 0.33^a^ |
|  | Middle | 2.23±1.38^a^ | 1.74±1.71^a^ | 0.88±0.74^a^ | 1.80±1.36^a^ | 0.53±0.07^a^ | 0.41^a^ |
|  | Total | 2.71±1.78^a^ | 2.02±2.27^a^ | 0.98±0.97^a^ | 1.82±1.27^a^ | 0.44±0.12^a^ | 0.37^a^ |
| Myosmine | Upper | 0.07±0.04^a^ | 0.07±0.06^a^ | 0.08±0.03^a^ | 0.11±0.05^b^ | 0.05±0.01^bc^ | 0.04^bc^ |
|  | Middle | 0.06±0.04^b^ | 0.07±0.06^b^ | 0.06±0.03^b^ | 0.11±0.05^a^ | 0.05±0.01^b^ | 0.05^b^ |
|  | Total | 0.06±0.04^a^ | 0.07±0.06^a^ | 0.07±0.03^a^ | 0.11±0.05^a^ | 0.05±0.01^a^ | 0.05^a^ |
| Anabasine | Upper | 0.25±0.03^a^ | 0.23±0.07^ab^ | 0.14±0.02^b^ | 0.13±0.02^b^ | 0.15±0.02^b^ | 0.13^b^ |
|  | Middle | 0.17±0.04^a^ | 0.20±0.03^a^ | 0.14±0.02^b^ | 0.12±0.01^b^ | 0.13±0.00^b^ | 0.16^a^ |
|  | Total | 0.21±0.06^a^ | 0.21±0.06^a^ | 0.14±0.02^a^ | 0.12±0.02^a^ | 0.14±0.02^a^ | 0.14^a^ |
| Anatabine | Upper | 2.31±0.43^a^ | 1.73±1.03^a^ | 0.64±0.21^a^ | 0.55±0.24^a^ | 0.95±0.13^a^ | 0.62^a^ |
|  | Middle | 1.51±0.45^a^ | 1.55±0.49^a^ | 0.80±0.14^a^ | 0.52±0.15^a^ | 0.85±0.05^a^ | 1.24^a^ |
|  | Total | 1.91±0.60^a^ | 1.64±0.79^a^ | 0.72±0.18^a^ | 0.53±0.19^a^ | 0.90±0.10^a^ | 0.93^a^ |
| Total alkaloids | Upper | 47.51±10.25^a^ | 36.43±14.90^a^ | 29.60±5.22^a^ | 34.22±5.70^a^ | 30.95±1.22^a^ | 30.34^a^ |
|  | Middle | 31.59±8.50^a^ | 32.51±8.29^a^ | 31.72±1.96^a^ | 30.01±3.82^a^ | 28.87±1.72^a^ | 36.84^a^ |
|  | Total | 39.55±12.27^a^ | 34.47±11.87^a^ | 30.66±3.82^a^ | 32.12±5.08^a^ | 29.91±1.71^a^ | 33.59^a^ |

Data are the means ± standard deviation

Values in the same row with different letters indicate significant difference (*P*<0.05) according to Duncan’s tests

The total represents a mixture of upper and middle leaves

**Table S10** The polyacids and higher fatty acids contents in cigar tobaccos of different origins (mg/g).

| Indices | Parts | Guangcun | Changjiang | Baisha | Datian | Wuzhishan | Chengmai |
| --- | --- | --- | --- | --- | --- | --- | --- |
| Oxalic acid | Upper | 31.60±4.39^a^ | 29.80±7.09^a^ | 29.99±4.46^a^ | 28.92±4.36^a^ | 28.58±0.35^a^ | 26.66^a^ |
|  | Middle | 25.46±3.28^a^ | 25.87±3.70^a^ | 26.85±3.32^a^ | 24.88±4.10^a^ | 29.28±0.19^a^ | 24.95^a^ |
|  | Total | 28.53±4.91^a^ | 27.84±5.84^a^ | 28.42±4.01^a^ | 26.9±4.52^a^ | 28.93±0.46^a^ | 25.81^a^ |
| Malonic acid | Upper | 1.56±0.66^a^ | 1.99±0.34^a^ | 2.23±0.26^a^ | 1.87±0.31^a^ | 2.15±0.34^a^ | 1.72^a^ |
|  | Middle | 1.83±0.46^a^ | 2.00±0.31^a^ | 2.10±0.53^a^ | 2.03±0.24^a^ | 1.94±0.16^a^ | 1.49^a^ |
|  | Total | 1.70±0.57^a^ | 1.99±0.32^a^ | 2.17±0.39^a^ | 1.95±0.27^a^ | 2.04±0.25^a^ | 1.61^a^ |
| Succinic acid | Upper | 0.52±0.10^a^ | 1.93±2.17^b^ | 1.28±1.54^a^ | 0.79±0.57^a^ | 0.62±0.23^a^ | 0.31^a^ |
|  | Middle | 0.42±0.09^a^ | 2.14±3.00^b^ | 0.57±0.10^a^ | 1.65±1.73^a^ | 0.49±0.03^a^ | 0.56^a^ |
|  | Total | 0.47±0.10^a^ | 2.03±2.54^b^ | 0.92±1.08^a^ | 1.22±1.30^a^ | 0.56±0.15^a^ | 0.44^a^ |
| Malic acid | Upper | 55.94±9.90^a^ | 47.39±23.80^a^ | 47.06±20.29^a^ | 29.69±12.30^ab^ | 36.43±15.00^ab^ | 10.59^b^ |
|  | Middle | 49.34±8.88^b^ | 50.55±19.01^b^ | 58.06±18.36^a^ | 30.87±8.95^c^ | 47.78±4.81^b^ | 37.57^b^ |
|  | Total | 52.64±9.73^a^ | 48.97±20.96^a^ | 52.56±18.85^a^ | 30.28±10.16^a^ | 42.1±11.21^a^ | 24.08^a^ |
| Palmitic acid | Upper | 0.66±0.11^a^ | 0.58±0.10^ab^ | 0.43±0.04^bc^ | 0.39±0.05^c^ | 0.37±0.12^c^ | 0.49^bc^ |
|  | Middle | 0.69±0.14^ab^ | 0.61±0.12^ab^ | 0.45±0.05^b^ | 0.42±0.08^b^ | 0.47±0.01^b^ | 0.48^b^ |
|  | Total | 0.67±0.13^a^ | 0.59±0.11^a^ | 0.44±0.04^a^ | 0.41±0.06^a^ | 0.42±0.09^a^ | 0.49^a^ |
| Citric acid | Upper | 15.81±5.97^a^ | 20.67±14.28^a^ | 14.50±6.14^a^ | 15.03±3.30^a^ | 16.99±3.85^a^ | 10.48^a^ |
|  | Middle | 16.54±5.38^a^ | 21.42±16.22^a^ | 17.97±6.00^a^ | 17.78±4.87^a^ | 15.25±0.18^a^ | 22.08^a^ |
|  | Total | 16.18±5.52^a^ | 21.05±14.83^a^ | 16.23±5.92^a^ | 16.41±4.18^a^ | 16.12±2.44^a^ | 16.28^a^ |
| Oleic acid | Upper | 0.24±0.08^ab^ | 0.19±0.05^abc^ | 0.17±0.05^abc^ | 0.26±0.05^a^ | 0.13±0.01^bc^ | 0.12^a^ |
|  | Middle | 0.20±0.06^ab^ | 0.18±0.04^ab^ | 0.16±0.05^ab^ | 0.23±0.06^a^ | 0.19±0.07^ab^ | 0.12^b^ |
|  | Total | 0.22±0.07^a^ | 0.18±0.05^a^ | 0.16±0.04^a^ | 0.25±0.05^a^ | 0.16±0.05^a^ | 0.12^a^ |
| Linoleic acid | Upper | 0.52±0.16^a^ | 0.33±0.10^ab^ | 0.28±0.09^b^ | 0.26±0.08^b^ | 0.34±0.11^ab^ | 0.34^ab^ |
|  | Middle | 0.42±0.19^a^ | 0.30±0.08^a^ | 0.25±0.06^a^ | 0.24±0.06^a^ | 0.34±0.01^a^ | 0.30^a^ |
|  | Total | 0.47±0.18^a^ | 0.31±0.09^a^ | 0.27±0.07^a^ | 0.25±0.07^a^ | 0.34±0.07^a^ | 0.32^a^ |
| Linolenic acid | Upper | 0.49±0.30^a^ | 0.27±0.16^a^ | 0.15±0.04^a^ | 0.26±0.16^a^ | 0.42±0.23^a^ | 0.52^a^ |
|  | Middle | 0.40±0.23^a^ | 0.27±0.17^a^ | 0.20±0.04^a^ | 0.28±0.15^a^ | 0.36±0.04^a^ | 0.42^a^ |
|  | Total | 0.44±0.26^a^ | 0.27±0.16^a^ | 0.17±0.05^a^ | 0.27±0.15^a^ | 0.39±0.14^a^ | 0.47^a^ |
| Total polyacids | Upper | 107.35±9.35^a^ | 103.13±25.90^a^ | 96.08±28.69^a^ | 77.46±9.74^b^ | 86.02±19.10^b^ | 51.24^c^ |
|  | Middle | 95.30±14.52^a^ | 103.34±15.10^a^ | 106.61±17.28^a^ | 78.39±7.08^b^ | 96.08±4.65^a^ | 87.96^ab^ |
|  | Total | 101.30±13.37^a^ | 103.24±20.50^a^ | 101.34±22.64^a^ | 77.93±9.73^a^ | 91.05±12.75^a^ | 69.60^a^ |

Data are the means ± standard deviation

Values in the same row with different letters indicate significant difference (*P*<0.05) according to Duncan’s tests

The total represents a mixture of upper and middle leaves

**Table S11** The volatile acids contents in cigar tobaccos of different origins (μg/g).

| Indices | Parts | Guangcun | Changjiang | Baisha | Datian | Wuzhishan | Chengmai |
| --- | --- | --- | --- | --- | --- | --- | --- |
| Acetic acid | Upper | 46.25±13.65^a^ | 66.14±48.09^a^ | 35.84±20.76^a^ | 24.99±3.67^a^ | 30.43±2.59^a^ | 53.30^a^ |
|  | Middle | 45.68±17.56^a^ | 68.57±77.71^a^ | 19.27±7.01^a^ | 35.85±25.77^a^ | 29.27±7.25^a^ | 33.42^a^ |
|  | Total | 45.97±15.26^a^ | 67.35±62.70^a^ | 27.55±16.86^a^ | 30.42±18.27^a^ | 29.85±4.50^a^ | 43.36^a^ |
| Formic acid | Upper | 89.56±4.07^a^ | 91.46±9.05^a^ | 86.42±2.03^a^ | 87.67±0.26^a^ | 87.93±0.02^a^ | 89.57^a^ |
|  | Middle | 87.86±2.93^a^ | 92.97±11.89^a^ | 87.48±1.93^a^ | 87.35±0.47^a^ | 87.68±0.50^a^ | 88.79^a^ |
|  | Total | 88.71±3.55^a^ | 92.22±10.28^a^ | 86.95±1.92^a^ | 87.51±0.40^a^ | 87.8±0.32^a^ | 89.18^a^ |
| Propionic acid | Upper | 1.12±0.52^a^ | 1.75±1.16^a^ | 0.94±0.65^a^ | 0.48±0.20^a^ | 0.96±0.11^a^ | 1.30^a^ |
|  | Middle | 1.22±0.68^a^ | 1.83±1.14^a^ | 0.49±0.50^a^ | 0.80±0.37^a^ | 0.85±0.23^a^ | 0.56^a^ |
|  | Total | 1.17±0.59^a^ | 1.79±1.12^a^ | 0.71±0.59^a^ | 0.64±0.33^a^ | 0.91±0.16^a^ | 0.93^a^ |
| Isobutyric acid | Upper | 2.86±2.03^a^ | 4.10±3.23^a^ | 1.52±0.51^a^ | 1.44±0.29^a^ | 1.74±0.68^a^ | 2.47^a^ |
|  | Middle | 1.81±1.71^a^ | 3.11±2.04^a^ | 0.96±0.37^a^ | 1.61±0.86^a^ | 2.44±1.40^a^ | 0.87^a^ |
|  | Total | 2.33±1.90^a^ | 3.60±2.67^a^ | 1.24±0.51^a^ | 1.52±0.61^a^ | 2.09±0.99^a^ | 1.67^a^ |
| Butyric acid | Upper | 1.47±0.95^a^ | 1.07±0.52^a^ | 0.59±0.12^a^ | 0.47±0.09^a^ | 0.51±0.30^a^ | 0.69^a^ |
|  | Middle | 1.14±1.38^a^ | 0.98±0.47^a^ | 0.39±0.20^a^ | 0.59±0.16^a^ | 0.97±0.60^a^ | 0.41^a^ |
|  | Total | 1.31±1.16^a^ | 1.02±0.48^a^ | 0.49±0.18^a^ | 0.53±0.14^a^ | 0.74±0.47^a^ | 0.55^a^ |
| Isovaleric acid | Upper | 31.33±13.20^a^ | 32.15±24.48^a^ | 13.96±3.91^a^ | 22.02±3.54^a^ | 16.03±5.11^a^ | 32.96^a^ |
|  | Middle | 21.69±15.98 | 26.36±16.32^a^ | 11.20±4.16^a^ | 20.84±7.49^a^ | 22.30±10.49^a^ | 9.65^a^ |
|  | Total | 26.51±15.06^a^ | 29.25±20.40^a^ | 12.58±4.02^a^ | 21.43±5.56^a^ | 19.19±7.66^a^ | 21.31^a^ |
| Valeric acid | Upper | 0.91±0.32^a^ | 0.85±0.32^a^ | 0.51±0.13^a^ | 0.55±0.08^a^ | 0.47±0.18^a^ | 0.77^a^ |
|  | Middle | 0.81±0.52^a^ | 0.79±0.35^a^ | 0.42±0.20^a^ | 0.60±0.15^a^ | 0.66±0.17^a^ | 0.53^a^ |
|  | Total | 0.86±0.42^a^ | 0.82±0.33^a^ | 0.46±0.17^a^ | 0.57±0.12^a^ | 0.56±0.18^a^ | 0.65^a^ |
| Hexanoic acid | Upper | 7.52±3.02^a^ | 2.14±3.38^a^ | 0.27±0.12^a^ | 0.26±0.06^a^ | 0.22±0.02^a^ | 0.24^a^ |
|  | Middle | 3.12±4.09^a^ | 0.94±1.69^a^ | 0.21±0.14^a^ | 0.22±0.03^a^ | 0.23±0.01^a^ | 0.23^a^ |
|  | Total | 5.32±4.16^a^ | 1.54±2.66^a^ | 0.24±0.12^a^ | 0.24±0.05^a^ | 0.22±0.01^a^ | 0.24^a^ |
| Octanoic acid | Upper | 0.27±0.11^a^ | 1.15±0.93^a^ | 1.82±0.58^a^ | 1.62±0.25^a^ | 1.45±0.40^a^ | 1.65^a^ |
|  | Middle | 0.61±1.23^a^ | 0.94±0.75^a^ | 1.33±0.76^a^ | 1.43±0.40^a^ | 1.63±0.34^a^ | 1.48^a^ |
|  | Total | 0.44±0.87^a^ | 1.04±0.83^a^ | 1.58±0.68^a^ | 1.53±0.33^a^ | 1.54±0.32^a^ | 1.57^a^ |
| Nonanoic acid | Upper | 0.55±0.17^b^ | 1.12±0.51^ab^ | 1.61±0.48^a^ | 1.62±0.53^a^ | 1.08±0.06^a^ | 1.86^a^ |
|  | Middle | 1.56±2.55^a^ | 1.21±0.71^a^ | 1.21±0.45^a^ | 1.25±0.70^a^ | 2.14±1.10^a^ | 2.32^a^ |
|  | Total | 1.06±1.83^a^ | 1.16±0.60^a^ | 1.41±0.48^a^ | 1.44±0.62^a^ | 1.61±0.89^a^ | 2.09^a^ |
| Total | Upper | 181.84±32.10^a^ | 201.92±65.73^c^ | 143.48±18.86^b^ | 141.12±6.83^b^ | 140.8±9.31^b^ | 184.81^a^ |
|  | Middle | 165.50±37.78^a^ | 197.69±91.68^a^ | 122.95±11.79^a^ | 150.55±32.65^a^ | 148.20±21.40^a^ | 138.26^a^ |
|  | Total | 173.67±35.10^a^ | 199.80±77.41^a^ | 133.21±18.23^a^ | 145.84±22.79^a^ | 144.50±14.14^a^ | 161.54^a^ |

Data are the means ± standard deviation

Values in the same row with different letters indicate significant difference (*P*<0.05) according to Duncan’s tests

The total represents a mixture of upper and middle leaves

**Table S12** The sugar alcohols contents in cigar tobaccos of different origins (mg/g).

| Indices | Parts | Guangcun | Changjiang | Baisha | Datian | Wuzhishan | Chengmai |
| --- | --- | --- | --- | --- | --- | --- | --- |
| Propanetriol | Upper | 0.60±0.34^a^ | 0.31±0.18^a^ | 0.19±0.11^a^ | 0.23±0.17^a^ | 0.30±0.11^a^ | 0.15^a^ |
|  | Middle | 0.73±0.44^a^ | 0.35±0.21^ab^ | 0.30±0.11^ab^ | 0.22±0.22^b^ | 0.31±0.04^ab^ | 0.18^b^ |
|  | Total | 0.66±0.39^a^ | 0.33±0.19^a^ | 0.25±0.12^a^ | 0.23±0.19^a^ | 0.30±0.06^a^ | 0.17^a^ |
| Mannitol | Upper | 0.18±0.05^a^ | 0.12±0.06^a^ | 0.15±0.08^a^ | 0.42±0.32^ab^ | 0.35±0.13^ab^ | 0.09^b^ |
|  | Middle | 0.16±0.05^a^ | 0.13±0.06^a^ | 0.17±0.04^a^ | 0.20±0.08^a^ | 0.22±0.00^a^ | 0.11^a^ |
|  | Total | 0.17±0.05^a^ | 0.12±0.06^a^ | 0.16±0.06^a^ | 0.31±0.25^a^ | 0.28±0.11^a^ | 0.10^a^ |
| Mannose | Upper | 0.52±0.43^ab^ | 0.32±0.32^ab^ | 0.21±0.11^ab^ | 0.09±0.01^a^ | 0.14±0.00^b^ | 0.13^b^ |
|  | Middle | 0.77±1.00^a^ | 0.42±0.72^a^ | 0.20±0.03^a^ | 0.11±0.04^a^ | 0.20±0.08^a^ | 0.09^a^ |
|  | Total | 0.64±0.76^a^ | 0.37±0.54^a^ | 0.21±0.07^a^ | 0.10±0.03^a^ | 0.17±0.06^a^ | 0.11^a^ |
| Fructose | Upper | 0.72±0.63^a^ | 0.46±0.47^a^ | 0.28±0.17^a^ | 0.11±0.03^a^ | 0.19±0.01^a^ | 0.19^a^ |
|  | Middle | 1.15±1.56^a^ | 0.60±1.05^a^ | 0.26±0.05^a^ | 0.15±0.05^a^ | 0.27±0.13^a^ | 0.12^a^ |
|  | Total | 0.93±1.17^a^ | 0.53±0.79^b^ | 0.27±0.11^b^ | 0.13±0.04^b^ | 0.23±0.09^b^ | 0.16^b^ |
| Inositol | Upper | 1.65±1.11^a^ | 1.27±0.88^a^ | 0.86±0.12^a^ | 0.42±0.10^a^ | 0.82±0.41^a^ | 0.48^a^ |
|  | Middle | 1.18±0.68^a^ | 1.47±0.98^a^ | 1.13±0.72^a^ | 0.52±0.16^a^ | 0.62±0.04^a^ | 0.73^a^ |
|  | Total | 1.41±0.92^a^ | 1.37±0.91^a^ | 0.99±0.50^a^ | 0.47±0.13^a^ | 0.72±0.26^a^ | 0.61^a^ |
| Xylitol | Upper | 0.16±0.05^a^ | 0.10±0.06^a^ | 0.09±0.06^a^ | 0.19±0.13^a^ | 0.17±0.06^a^ | 0.14^a^ |
|  | Middle | 0.12±0.05^a^ | 0.11±0.06^a^ | 0.13±0.02^a^ | 0.13±0.06^a^ | 0.12±0.04^a^ | 0.14^a^ |
|  | Total | 0.14±0.05^a^ | 0.10±0.06^a^ | 0.11±0.04^a^ | 0.16±0.10^a^ | 0.14±0.05^a^ | 0.14^a^ |
| Raffinose  （μg/g） | Upper | 9.40±4.99^a^ | 10.65±11.38^a^ | 6.48±1.31^a^ | 6.29±2.12^a^ | 4.89±1.23^a^ | 7.38^a^ |
|  | Middle | 11.11±3.43^a^ | 10.08±12.03^a^ | 8.42±3.43^a^ | 6.40±1.26^a^ | 4.89±0.09^a^ | 5.84^a^ |
|  | Total | 10.25±4.25^a^ | 10.36±11.36^a^ | 7.45±2.62^a^ | 6.35±1.64^a^ | 4.89±0.71^a^ | 6.61^a^ |
| Glucose | Upper | 0.79±0.68^a^ | 0.50±0.51^a^ | 0.31±0.17^a^ | 0.12±0.02^a^ | 0.21±0.02^a^ | 0.22^a^ |
|  | Middle | 1.27±1.74^a^ | 0.68±1.17^b^ | 0.29±0.06^b^ | 0.16±0.05^b^ | 0.35±0.18^b^ | 0.13^b^ |
|  | Total | 1.03±1.30^a^ | 0.59±0.88^b^ | 0.30±0.12^b^ | 0.14±0.04^b^ | 0.28±0.13^b^ | 0.18^b^ |
| Rhamnose  （μg/g） | Upper | 35.22±17.00^a^ | 70.86±45.10^a^ | 61.74±35.59^a^ | 65.37±21.96^a^ | 81.79±27.28^a^ | 46.92^a^ |
|  | Middle | 30.86±19.96^a^ | 41.93±28.48^a^ | 36.49±18.77^a^ | 44.29±17.25^a^ | 36.60±15.56^a^ | 34.89^a^ |
|  | Total | 33.04±18.13^a^ | 56.39±39.50^a^ | 49.11±29.60^a^ | 54.83±21.68^a^ | 59.19±31.78^a^ | 40.91^a^ |
| Sucrose | Upper | 0.12±0.09^a^ | 0.14±0.20^a^ | 0.08±0.01^a^ | 0.04±0.02^a^ | 0.06±0.01^a^ | 0.06^a^ |
|  | Middle | 0.15±0.10^a^ | 0.20±0.37^a^ | 0.09±0.02^a^ | 0.05±0.02^a^ | 0.08±0.04^a^ | 0.03^a^ |
|  | Total | 0.13±0.09^a^ | 0.17±0.29^a^ | 0.09±0.02^a^ | 0.04±0.02^a^ | 0.07±0.03^a^ | 0.05^a^ |
| Total | Upper | 4.78±2.11^a^ | 3.31±2.19^a^ | 2.24±0.74^a^ | 1.69±0.63^a^ | 2.31±0.66^a^ | 1.51^a^ |
|  | Middle | 5.57±4.66^a^ | 4.01±3.56^a^ | 2.61±0.72^a^ | 1.59±0.47^a^ | 2.19±0.48^a^ | 1.57^a^ |
|  | Total | 5.18±3.53^a^ | 3.66±2.89^a^ | 2.42±0.71^a^ | 1.64±0.53^a^ | 2.25±0.48^a^ | 1.54^a^ |

Data are the means ± standard deviation

Values in the same row with different letters indicate significant difference (*P*<0.05) according to Duncan’s tests

The total represents a mixture of upper and middle leaves

**Table S13** The amino acids contents in cigar tobaccos of different origins (mg/g).

| Indices | Parts | Guangcun | Changjiang | Baisha | Datian | Wuzhishan | Chengmai |
| --- | --- | --- | --- | --- | --- | --- | --- |
| Ala | Upper | 0.51±0.23^a^ | 0.37±0.25^a^ | 0.11±0.06^ab^ | 0.12±0.09^ab^ | 0.31±0.01^a^ | 0.11^b^ |
|  | Middle | 0.28±0.14^a^ | 0.31±0.18^a^ | 0.19±0.10^a^ | 0.14±0.12^a^ | 0.28±0.02^a^ | 0.15^a^ |
|  | Total | 0.39±0.22^a^ | 0.34±0.21^a^ | 0.15±0.09^a^ | 0.13±0.10^a^ | 0.30±0.02^a^ | 0.13^a^ |
| Arg | Upper | 0.06±0.03^a^ | 0.05±0.03^ab^ | 0.02±0.01^b^ | 0.03±0.02^b^ | 0.06±0.01^a^ | 0.04^b^ |
|  | Middle | 0.05±0.02^a^ | 0.05±0.03^a^ | 0.03±0.00^a^ | 0.03±0.01^a^ | 0.06±0.01^a^ | 0.04^a^ |
|  | Total | 0.06±0.03^a^ | 0.05±0.03^a^ | 0.03±0.01^a^ | 0.03±0.02^a^ | 0.06±0.01^a^ | 0.04^a^ |
| Asn | Upper | 1.58±1.69^a^ | 0.73±1.23^a^ | 0.22±0.33^a^ | 0.26±0.21^a^ | 0.19±0.10^a^ | 1.56^a^ |
|  | Middle | 1.15±1.22^a^ | 0.53±0.84^a^ | 0.48±0.31^a^ | 0.35±0.31^a^ | 1.08±0.48^a^ | 0.99^a^ |
|  | Total | 1.37±1.45^a^ | 0.63±1.03^a^ | 0.35±0.33^a^ | 0.31±0.25^a^ | 0.64±0.58^a^ | 1.28^a^ |
| Asp | Upper | 8.40±2.85^a^ | 7.85±3.66^a^ | 5.33±2.89^a^ | 8.83±5.94^a^ | 12.66±2.74^a^ | 8.83^a^ |
|  | Middle | 9.13±2.94^a^ | 9.84±3.76^a^ | 8.22±1.21^a^ | 10.81±4.46^a^ | 13.41±2.24^a^ | 9.84^a^ |
|  | Total | 8.76±2.84^a^ | 8.85±3.74^a^ | 6.78±2.57^a^ | 9.82±5.06^a^ | 13.04±2.09^a^ | 9.34^a^ |
| Cys  (μg/g) | Upper | 5.72±0.09^a^ | 5.60±0.03^a^ | 5.65±0.07^a^ | 5.58±0.04^a^ | 5.62±0.02^a^ | 5.60^a^ |
|  | Middle | 5.72±0.14^a^ | 5.66±0.06^a^ | 5.64±0.06^a^ | 5.61±0.05^a^ | 5.68±0.09^a^ | 5.58^a^ |
|  | Total | 5.72±0.11^a^ | 5.63±0.05^a^ | 5.64±0.06^a^ | 5.60±0.05^a^ | 5.65±0.06^a^ | 5.59^a^ |
| Gln | Upper | 0.31±0.15^a^ | 0.22±0.14^b^ | 0.07±0.06^ab^ | 0.13±0.09^b^ | 0.25±0.09^a^ | 0.18^b^ |
|  | Middle | 0.22±0.09^a^ | 0.21±0.11^a^ | 0.12±0.02^a^ | 0.13±0.07^a^ | 0.24±0.03^a^ | 0.19^a^ |
|  | Total | 0.26±0.13^a^ | 0.21±0.12^a^ | 0.10±0.05^a^ | 0.13±0.08^a^ | 0.25±0.05^a^ | 0.19^a^ |
| Glu | Upper | 0.99±0.52^a^ | 0.84±0.60^a^ | 0.40±0.19^ab^ | 0.49±0.36^ab^ | 1.29±0.31^b^ | 0.55^ab^ |
|  | Middle | 0.98±0.35^a^ | 0.99±0.65^a^ | 0.72±0.22^a^ | 0.50±0.25^a^ | 0.89±0.01^a^ | 0.63^a^ |
|  | Total | 0.98±0.43^a^ | 0.92±0.61^a^ | 0.56±0.26^a^ | 0.50±0.29^a^ | 1.09±0.29^a^ | 0.59^a^ |
| Gly | Upper | 0.16±0.05^a^ | 0.10±0.05^a^ | 0.06±0.03^a^ | 0.06±0.04^a^ | 0.14±0.02^a^ | 0.06^a^ |
|  | Middle | 0.11±0.05^a^ | 0.09±0.05^a^ | 0.09±0.04^a^ | 0.05±0.06^a^ | 0.13±0.02^a^ | 0.08^a^ |
|  | Total | 0.14±0.06^a^ | 0.10±0.05^a^ | 0.07±0.03^a^ | 0.06±0.05^a^ | 0.13±0.02^a^ | 0.07^a^ |
| His | Upper | 0.28±0.12^a^ | 0.18±0.06^b^ | 0.15±0.05^b^ | 0.17±0.09^b^ | 0.18±0.05^b^ | 0.15^b^ |
|  | Middle | 0.24±0.11^a^ | 0.17±0.06^a^ | 0.16±0.02^a^ | 0.14±0.05^a^ | 0.25±0.05^a^ | 0.15^a^ |
|  | Total | 0.26±0.12^a^ | 0.18±0.06^a^ | 0.15±0.03^a^ | 0.16±0.07^a^ | 0.21±0.06^a^ | 0.15^a^ |
| Ile | Upper | 0.09±0.05^a^ | 0.07±0.04^a^ | 0.03±0.01^a^ | 0.03±0.02^a^ | 0.08±0.02^a^ | 0.03^a^ |
|  | Middle | 0.05±0.03^a^ | 0.07±0.03^a^ | 0.04±0.01^a^ | 0.03±0.02^a^ | 0.06±0.01^a^ | 0.04^a^ |
|  | Total | 0.07±0.05^a^ | 0.07±0.04^a^ | 0.03±0.01^a^ | 0.03±0.02^a^ | 0.07±0.02^a^ | 0.03^a^ |
| Leu | Upper | 0.17±0.09^a^ | 0.13±0.08^a^ | 0.04±0.03^a^ | 0.06±0.04^a^ | 0.16±0.08^a^ | 0.06^a^ |
|  | Middle | 0.10±0.05^a^ | 0.12±0.06^a^ | 0.07±0.03^a^ | 0.06±0.05^a^ | 0.13±0.02^a^ | 0.08^a^ |
|  | Total | 0.14±0.08^a^ | 0.13±0.07^a^ | 0.05±0.03^a^ | 0.06±0.04^a^ | 0.15±0.05^a^ | 0.07^a^ |
| lys | Upper | 0.15±0.08^a^ | 0.10±0.07^a^ | 0.03±0.02^a^ | 0.06±0.04^a^ | 0.13±0.06^a^ | 0.10^a^ |
|  | Middle | 0.11±0.05^a^ | 0.11±0.06^a^ | 0.05±0.01^a^ | 0.06±0.04^a^ | 0.13±0.03^a^ | 0.09^a^ |
|  | Total | 0.13±0.07^a^ | 0.10±0.06^a^ | 0.04±0.02^a^ | 0.06±0.04^a^ | 0.13±0.04^a^ | 0.10^a^ |
| Phe | Upper | 0.39±0.25^a^ | 0.20±0.18^a^ | 0.04±0.04^a^ | 0.14±0.14^a^ | 0.26±0.20^a^ | 0.19^a^ |
|  | Middle | 0.26±0.19^a^ | 0.18±0.15^a^ | 0.07±0.02^a^ | 0.14±0.14^a^ | 0.31±0.18^a^ | 0.18^a^ |
|  | Total | 0.33±0.23^a^ | 0.19±0.16^a^ | 0.06±0.03^a^ | 0.14±0.13^a^ | 0.28±0.16^a^ | 0.19^a^ |
| Pro | Upper | 1.55±0.84^a^ | 1.15±0.77^a^ | 0.29±0.14^a^ | 0.32±0.35^a^ | 0.75±0.53^a^ | 0.23^a^ |
|  | Middle | 1.09±0.59^a^ | 1.06±0.60^a^ | 0.51±0.34^a^ | 0.35±0.32^a^ | 0.70±0.03^a^ | 0.34^a^ |
|  | Total | 1.32±0.74^a^ | 1.10±0.67^a^ | 0.40±0.27^a^ | 0.33±0.31^a^ | 0.72±0.31^a^ | 0.28^a^ |
| Ser | Upper | 0.32±0.20^a^ | 0.19±0.13^ab^ | 0.05±0.02^b^ | 0.07±0.05^b^ | 0.18±0.05^ab^ | 0.16^b^ |
|  | Middle | 0.29±0.15^a^ | 0.23±0.16^a^ | 0.13±0.06^a^ | 0.08±0.06^a^ | 0.24±0.12^a^ | 0.20^a^ |
|  | Total | 0.30±0.17^a^ | 0.21±0.14^a^ | 0.09±0.06^a^ | 0.08±0.05^a^ | 0.21±0.08^a^ | 0.18^a^ |
| Thr | Upper | 0.58±0.22^a^ | 0.36±0.17^ab^ | 0.25±0.09^b^ | 0.36±0.09^b^ | 0.49±0.17^ab^ | 0.51^b^ |
|  | Middle | 0.51±0.17^a^ | 0.39±0.15^a^ | 0.31±0.07^a^ | 0.31±0.10^a^ | 0.49±0.11^a^ | 0.52^a^ |
|  | Total | 0.54±0.19^a^ | 0.38±0.15^a^ | 0.28±0.08^a^ | 0.34±0.09^a^ | 0.49±0.12^a^ | 0.52^a^ |
| Trp | Upper | 0.11±0.11^a^ | 0.05±0.05^ab^ | 0.02±0.02^b^ | 0.05±0.06^ab^ | 0.10±0.07^b^ | 0.07^b^ |
|  | Middle | 0.10±0.08^a^ | 0.05±0.05^a^ | 0.03±0.02^a^ | 0.04±0.03^a^ | 0.11±0.05^a^ | 0.06^a^ |
|  | Total | 0.11±0.10^a^ | 0.05±0.05^a^ | 0.03±0.02^a^ | 0.05±0.05^a^ | 0.11±0.05^a^ | 0.07^a^ |
| Tyr | Upper | 0.08±0.04^a^ | 0.05±0.04^a^ | 0.01±0.01^a^ | 0.04±0.03^a^ | 0.07±0.06^a^ | 0.02^a^ |
|  | Middle | 0.06±0.04^a^ | 0.05±0.03^a^ | 0.02±0.00^a^ | 0.03±0.02^a^ | 0.07±0.03^a^ | 0.02^a^ |
|  | Total | 0.07±0.04^a^ | 0.05±0.04^a^ | 0.02±0.01^a^ | 0.04±0.03^a^ | 0.07±0.05^a^ | 0.02^a^ |
| Val | Upper | 0.28±0.17^a^ | 0.25±0.16^a^ | 0.06±0.02^a^ | 0.07±0.04^a^ | 0.19±0.05^a^ | 0.08^a^ |
|  | Middle | 0.20±0.13^a^ | 0.25±0.13^a^ | 0.10±0.04^a^ | 0.07±0.04^a^ | 0.17±0.01^a^ | 0.11^a^ |
|  | Total | 0.07±0.04^a^ | 0.05±0.04^a^ | 0.02±0.01^a^ | 0.03±0.03^a^ | 0.07±0.04^a^ | 0.02^a^ |
| Total | Upper | 15.72±5.64^a^ | 12.73±6.64^a^ | 7.07±3.86^a^ | 11.12±7.43^a^ | 17.26±3.93^a^ | 12.68^a^ |
|  | Middle | 14.69±4.48^a^ | 14.49±6.12^a^ | 11.19±0.91^a^ | 13.17±5.77^a^ | 18.50±3.37^a^ | 13.46^a^ |
|  | Total | 15.20±4.97^a^ | 13.61±6.26^a^ | 9.13±3.40^a^ | 12.15±6.36^a^ | 17.88±3.07^a^ | 13.07^a^ |

Data are the means ± standard deviation

Values in the same row with different letters indicate significant difference (*P*<0.05) according to Duncan’s tests

The total represents a mixture of upper and middle leaves

**Table S14** The Amadori compounds contents in cigar tobaccos of different origins (μg/g).

| Indices | Parts | Guangcun | Changjiang | Baisha | Datian | Wuzhishan | Chengmai |
| --- | --- | --- | --- | --- | --- | --- | --- |
| Fru-Ala | Upper | 45.47±11.25^a^ | 32.80±16.68^a^ | 27.04±11.99^a^ | 78.72±33.53^a^ | 50.73±22.51^a^ | 60.31^a^ |
|  | Middle | 32.89±8.52^b^ | 28.30±11.80^b^ | 36.96±12.52^b^ | 85.31±39.58^a^ | 53.19±18.17^ab^ | 52.29^ab^ |
|  | Total | 39.18±11.65^a^ | 30.55±14.20^a^ | 32.00±12.53^a^ | 82.01±34.76^a^ | 51.96±16.76^a^ | 56.30^a^ |
| Fru-Asn | Upper | 71.77±102.51^a^ | 21.07±15.83^b^ | 12.27±8.71^b^ | 6.83±2.26^b^ | 9.83±3.53^b^ | 14.09^b^ |
|  | Middle | 89.24±136.70^a^ | 25.02±43.18^b^ | 10.15±3.34^b^ | 7.05±1.60^b^ | 19.92±9.15^b^ | 13.10^b^ |
|  | Total | 80.50±117.56^a^ | 23.05±31.62^a^ | 11.21±6.21^a^ | 6.94±1.85^a^ | 14.87±8.13^a^ | 13.60^a^ |
| Fru-Glu | Upper | 40.37±16.77^a^ | 37.23±23.67^a^ | 17.45±7.29^a^ | 17.30±6.86^a^ | 34.59±7.21^a^ | 18.59^a^ |
|  | Middle | 49.05±27.21^a^ | 44.32±44.48^a^ | 21.85±9.59^a^ | 16.72±5.43^a^ | 25.81±5.31^a^ | 18.55^a^ |
|  | Total | 44.71±22.37^a^ | 40.77±34.76^a^ | 19.65±8.23^a^ | 17.01±5.84^a^ | 30.20±7.24^a^ | 18.57^a^ |
| Fru-Gly | Upper | 53.28±40.85^a^ | 31.66±35.97^a^ | 24.44±30.33^a^ | 22.01±21.80^a^ | 28.70±18.18^a^ | 17.99^a^ |
|  | Middle | 58.09±69.69^a^ | 22.55±21.30^a^ | 21.03±18.22^a^ | 17.26±14.42^a^ | 27.30±1.79^a^ | 32.89^a^ |
|  | Total | 55.69±55.47^a^ | 27.10±29.06^a^ | 22.74±23.23^a^ | 19.64±17.60^a^ | 28.00±10.58^a^ | 25.44^a^ |
| Fru-Leu | Upper | 3.63±3.58^a^ | 2.01±1.47^a^ | 1.24±1.74^a^ | 0.74±0.70^a^ | 1.82±1.44^a^ | 0.65^a^ |
|  | Middle | 3.26±4.34^a^ | 1.68±1.85^a^ | 0.66±0.51^a^ | 0.46±0.33^a^ | 1.08±0.28^a^ | 0.53^a^ |
|  | Total | 3.44±3.87^a^ | 1.85±1.63^a^ | 0.95±1.23^a^ | 0.60±0.54^a^ | 1.45±0.95^a^ | 0.59^a^ |
| Fru-IIe | Upper | 3.75±3.72^a^ | 2.10±1.51^a^ | 1.22±1.61^a^ | 0.78±0.71^a^ | 1.95±1.37^a^ | 0.61^a^ |
|  | Middle | 3.36±4.36^a^ | 1.67±1.50^a^ | 0.83±0.49^a^ | 0.55±0.46^a^ | 1.12±0.11^a^ | 0.52^a^ |
|  | Total | 3.55±3.93^a^ | 1.89±1.48^a^ | 1.02±1.12^a^ | 0.67±0.58^a^ | 1.53±0.93^a^ | 0.57^a^ |
| Fru-Phe | Upper | 17.76±18.09^a^ | 8.12±6.31^a^ | 3.68±2.01^a^ | 2.94±1.10^a^ | 6.38±3.97^a^ | 3.76^a^ |
|  | Middle | 13.90±11.10^a^ | 9.42±14.09^a^ | 3.96±0.76^a^ | 2.71±0.76^a^ | 5.57±2.64^a^ | 2.87^a^ |
|  | Total | 15.83±14.70^a^ | 8.77±10.61^a^ | 3.82±1.42^a^ | 2.82±0.90^a^ | 5.97±2.79^a^ | 3.32^a^ |
| Fru-Pro | Upper | 131.03±94.20^a^ | 99.56±89.11^a^ | 52.00±40.11^a^ | 33.24±20.12^a^ | 57.59±43.76^a^ | 30.96^a^ |
|  | Middle | 175.29±140.05^a^ | 119.24±224.99^a^ | 39.75±6.70^a^ | 28.19±7.09^a^ | 52.46±35.14^a^ | 22.14^a^ |
|  | Total | 153.16±118.01^a^ | 109.4±166.32^a^ | 45.87±27.42^a^ | 30.71±14.47^a^ | 55.02±32.53^a^ | 26.55^a^ |
| Fru-Trp | Upper | 8.49±6.83^a^ | 5.22±4.10^a^ | 2.45±1.16^a^ | 2.37±0.71^a^ | 3.87±2.08^a^ | 2.12^a^ |
|  | Middle | 8.19±5.72^a^ | 7.36±12.4^a^ | 2.76±0.80^a^ | 1.64±0.42^a^ | 4.17±2.26^a^ | 34.89^a^ |
|  | Total | 8.34±6.11^a^ | 6.29±9.03^a^ | 2.60±0.94^a^ | 2.00±0.67^a^ | 4.02±1.78^a^ | 1.75^a^ |
| Fru-Val | Upper | 3.91±1.87^a^ | 3.83±2.29^a^ | 2.32±1.56^a^ | 4.13±3.35^a^ | 8.41±3.72^a^ | 1.88^a^ |
|  | Middle | 3.90±2.30^a^ | 4.37±3.21^a^ | 2.18±0.65^a^ | 2.13±1.29^a^ | 5.01±1.83^a^ | 1.31^a^ |
|  | Total | 3.90±2.03^a^ | 4.10±2.72^a^ | 2.25±1.11^a^ | 3.13±2.61^a^ | 6.71±3.10^a^ | 1.60^a^ |
| GLU | Upper | 614.76±422.10^a^ | 404.65±305.33^a^ | 205.37±234.91^a^ | 136.49±98.43^a^ | 226.85±150.78^a^ | 138.99^a^ |
|  | Middle | 578.39±589.95^a^ | 382.09±410.15^a^ | 180.75±85.80^a^ | 124.62±98.13^a^ | 310.69±62.29^a^ | 197.96^a^ |
|  | Total | 596.58±497.97^a^ | 393.37±350.96^a^ | 193.06±164.25^a^ | 130.56±92.87^a^ | 268.77±105.90^a^ | 168.48^a^ |
| Total | Upper | 994.22±684.13^a^ | 648.26±457.60^a^ | 349.47±339.36^a^ | 305.55±180.57^a^ | 430.70±258.55^a^ | 289.95^a^ |
|  | Middle | 1015.55±959.73^a^ | 646.01±746.41^a^ | 320.85±125.00^a^ | 286.63±156.41^a^ | 506.28±102.59^a^ | 343.52^a^ |
|  | Total | 1004.89±808.59^a^ | 647.14±600.60^a^ | 335.16±237.25^a^ | 296.09±159.57^a^ | 468.49±166.42^a^ | 316.74^a^ |

Data are the means ± standard deviation

Values in the same row with different letters indicate significant difference (*P*<0.05) according to Duncan’s tests

The total represents a mixture of upper and middle leaves

**Table S15** The polyphenols contents in cigar tobaccos of different origins (mg/g).

| Indices | Parts | Guangcun | Changjiang | Baisha | Datian | Wuzhishan | Chengmai |
| --- | --- | --- | --- | --- | --- | --- | --- |
| Neochlorogenic acid | Upper | 0.02±0.01^a^ | 0.03±0.01^a^ | 0.02±0.01^a^ | 0.02±0.01^a^ | 0.02±0.01^a^ | 0.02^a^ |
|  | Middle | 0.02±0.01^a^ | 0.03±0.01^a^ | 0.02±0.01^a^ | 0.02±0.01^a^ | 0.02±0.01^a^ | 0.02^a^ |
|  | Total | 0.02±0.01^a^ | 0.03±0.01^a^ | 0.02±0.00^a^ | 0.02±0.01^a^ | 0.02±0.01^a^ | 0.02^a^ |
| Chlorogenic acid | Upper | 0.46±0.09^a^ | 0.44±0.12^a^ | 0.58±0.16^a^ | 0.49±0.17^a^ | 0.47±0.15^a^ | 0.48^a^ |
|  | Middle | 0.50±0.05^a^ | 0.39±0.06^a^ | 0.58±0.02^a^ | 0.49±0.07^a^ | 0.49±0.07^a^ | 0.46^a^ |
|  | Total | 0.48±0.07^a^ | 0.41±0.09^a^ | 0.58±0.09^a^ | 0.49±0.12^a^ | 0.48±0.11^a^ | 0.47^a^ |
| Cryptochlorogenic acid | Upper | 0.13±0.01^a^ | 0.11±0.05^a^ | 0.13±0.08^a^ | 0.13±0.07^a^ | 0.11±0.03^a^ | 0.13^a^ |
|  | Middle | 0.12±0.01^a^ | 0.13±0.01^a^ | 0.11±0.03^a^ | 0.13±0.05^a^ | 0.11±0.01^a^ | 0.11^a^ |
|  | Total | 0.13±0.01^a^ | 0.14±0.03^a^ | 0.12±0.06^a^ | 0.13±0.06^a^ | 0.11±0.02^a^ | 0.12^a^ |
| Scopoletin | Upper | 0.03±0.01^a^ | 0.04±0.01^a^ | 0.03±0.01^a^ | 0.03±0.01^a^ | 0.02±0.01^a^ | 0.03^a^ |
|  | Middle | 0.03±0.01^a^ | 0.04±0.01^a^ | 0.03±0.02^a^ | 0.03±0.01^a^ | 0.02±0.00^a^ | 0.03^a^ |
|  | Total | 0.03±0.01^a^ | 0.04±0.01^a^ | 0.03±0.02^a^ | 0.03±0.01^a^ | 0.02±0.01^a^ | 0.03^a^ |
| Rutin | Upper | 0.54±0.06^a^ | 0.42±0.02^a^ | 0.48±0.08^a^ | 0.50±0.11^a^ | 0.45±0.09^a^ | 0.50^a^ |
|  | Middle | 0.58±0.04^a^ | 0.47±0.14^a^ | 0.52±0.14^a^ | 0.50±0.15^a^ | 0.49±0.05^a^ | 0.48^a^ |
|  | Total | 0.56±0.05^a^ | 0.52±0.08^a^ | 0.50±0.11^a^ | 0.50±0.13^a^ | 0.47±0.07^a^ | 0.49^a^ |
| Total | Upper | 1.19±0.18^a^ | 1.05±0.21^a^ | 1.24±0.26^a^ | 1.16±0.20^a^ | 1.07±0.11^a^ | 1.15^a^ |
|  | Middle | 1.26±0.16^a^ | 1.18±0.12^a^ | 1.26±0.09^a^ | 1.20±0.18^a^ | 1.17±0.09^a^ | 1.09^a^ |
|  | Total | 1.21±0.13^a^ | 1.14±0.17^a^ | 1.25±0.18^a^ | 1.18±0.19^a^ | 1.12±0.10^a^ | 1.12^a^ |

Data are the means ± standard deviation

Values in the same row with different letters indicate significant difference (*P*<0.05) according to Duncan’s tests

The total represents a mixture of upper and middle leaves

**Table S16** Pearson correlation between aroma precursors and flavor characteristics of cigar tobaccos from different origins of middle leaves.

| Compounds | Nutty | Bean | Coffee | Woody | Fruity | Honey-  sweet | Milk | Floral | Resin | Roasted |
| --- | --- | --- | --- | --- | --- | --- | --- | --- | --- | --- |
| Nornicotine | -0.17 | -0.08 | 0.07 | 0.01 | 0.49* | 0.01 | -0.14 | 0.17 | 0.10 | 0.03 |
| Myosmine | -0.19 | -0.04 | 0.01 | -0.03 | 0.47* | -0.09 | -0.07 | 0.12 | 0.36 | 0.05 |
| Anabasine | 0.03 | -0.01 | 0.22 | 0.05 | 0.40* | 0.24 | -0.19 | 0.01 | -0.19 | -0.02 |
| Malonic acid | 0.04 | 0.24 | -0.07 | 0.06 | 0.53* | 0.06 | -0.09 | -0.07 | -0.09 | 0.09 |
| Succinic acid | 0.22 | -0.08 | 0.07 | -0.40* | -0.05 | -0.12 | -0.14 | -0.13 | -0.16 | 0.03 |
| Oleic acid | 0.16 | 0.28 | -0.52* | 0.06 | -0.01 | -0.28 | -0.13 | -0.12 | 0.19 | -0.24 |
| Linoleic acid | 0.17 | 0.35 | -0.28 | 0.48* | 0.29 | 0.29 | 0.07 | 0.15 | -0.30 | 0.06 |
| Linolenic acid | 0.21 | 0.40* | -0.15 | 0.46* | 0.27 | 0.28 | 0.08 | 0.03 | -0.13 | 0.16 |
| Formic acid | 0.43* | -0.04 | 0.30 | 0.02 | 0.05 | 0.15 | -0.15 | -0.18 | -0.09 | 0.17 |
| Butyric acid | -0.25 | -0.02 | -0.25 | 0.08 | -0.03 | 0.16 | 0.21 | 0.43* | -0.10 | -0.06 |
| Asn | 0.21 | 0.40* | -0.23 | 0.54* | 0.34 | 0.46* | 0.20 | 0.27 | -0.24 | 0.19 |
| His | 0.22 | 0.44* | -0.14 | 0.42* | 0.52* | 0.43* | 0.23 | 0.23 | -0.33 | 0.23 |
| Trp | 0.19 | 0.48* | -0.14 | 0.41* | 0.51* | 0.38* | 0.24 | 0.21 | -0.28 | 0.23 |
| Cys | 0.41* | 0.24 | -0.10 | 0.30 | 0.25 | 0.35 | 0.01 | -0.07 | -0.41* | 0.25 |
| Mannose | 0.51* | 0.13 | 0.025 | 0.29 | 0.20 | 0.32 | -0.11 | -0.04 | -0.28 | 0.22 |
| Fructose | 0.51* | 0.13 | 0.016 | 0.30 | 0.20 | 0.32 | -0.11 | -0.04 | -0.27 | 0.21 |
| Raffinose | 0.47* | -0.014 | 0.40* | 0.08 | 0.24 | 0.18 | -0.17 | -0.08 | -0.25 | 0.26 |
| Glucose | 0.50* | 0.13 | 0.017 | 0.30 | 0.20 | 0.32 | -0.11 | -0.04 | -0.27 | 0.22 |
| Rhamnose | 0.023 | -0.18 | -0.18 | -0.40* | -0.13 | -0.16 | -0.1 | -0.06 | -0.043 | -0.11 |
| Inositol | 0.15 | -0.08 | -0.05 | -0.20 | -0.06 | -0.16 | -0.29 | -0.29 | -0.17 | -0.41* |
| Fru-Asn | 0.50* | 0.22 | -0.10 | 0.43* | 0.26 | 0.40* | -0.07 | -0.03 | -0.24 | 0.24 |
| Fru-Glu | 0.44* | 0.10 | 0.08 | 0.10 | 0.24 | 0.26 | -0.13 | -0.08 | -0.30 | 0.14 |
| Fru-IIe | 0.49* | 0.24 | -0.12 | 0.42* | 0.27 | 0.33 | -0.14 | -0.13 | -0.25 | 0.20 |
| Fru-Phe | 0.52* | 0.20 | 0.09 | 0.25 | 0.40* | 0.40* | -0.02 | 0.01 | -0.32 | 0.24 |
| Fru-Pro | 0.53* | 0.12 | 0.12 | 0.22 | 0.27 | 0.35 | -0.03 | 0.03 | -0.31 | 0.22 |
| Fru-Trp | 0.49* | 0.13 | 0.18 | 0.14 | 0.30 | 0.30 | -0.03 | 0.02 | -0.30 | 0.21 |
| Fru-Val | 0.40* | 0.31 | -0.02 | 0.093 | 0.29 | 0.22 | -0.001 | -0.04 | -0.31 | 0.13 |
| GLU | 0.45* | 0.19 | -0.07 | 0.27 | 0.21 | 0.30 | -0.10 | -0.07 | -0.28 | 0.18 |
| Fru-Leu | 0.50* | 0.23 | -0.10 | 0.36 | 0.26 | 0.34 | -0.14 | -0.12 | -0.25 | 0.21 |
| Neochlorogenic acid | -0.78** | -0.34 | 0.71** | -0.40* | 0.32 | -0.59* | 0.17 | 0.33 | 0.18 | -0.23 |
| Chlorogenic acid | 0.47* | -0.22 | 0.20 | 0.32 | 0.30 | -0.13 | 0.17 | 0.18 | -0.24 | 0.28 |
| Cryptochlorogenic acid | -0.52* | -0.02 | -0.21 | -0.54* | 0.30 | -0.005 | -0.43* | -0.35 | -0.03 | -0.42* |
| Scopoletin | -0.45* | -0.14 | -0.45* | -0.28 | 0.31 | 0.29 | -0.39 | -0.20 | -0.15 | 0.12 |
| Rutin | 0.004 | -0.26 | -0.28 | -0.77** | 0.33 | -0.03 | -0.44* | -0.50* | -0.78** | -0.85** |

* and ** represented *P*<0.05 and *P*<0.01, respectively

**Table S17** Pearson correlation between aroma precursors and style characteristics of cigar tobaccos from different origins of middle leaves.

| Compounds | Protein | Green | Burnt | Bitterness | Sweetness | Spicy | Astringent | Smoke concentration | Smoke strength | Transmissibility | Mellowness | Cigar-style manifestation |
| --- | --- | --- | --- | --- | --- | --- | --- | --- | --- | --- | --- | --- |
| Nicotine | 0.18 | 0.16 | -0.14 | 0.09 | -0.11 | 0.21 | 0.01 | 0.45* | 0.35 | 0.05 | -0.05 | 0.14 |
| Nornicotine | -0.41* | -0.17 | 0.12 | -0.11 | 0.06 | -0.13 | -0.09 | -0.15 | -0.08 | 0.34 | 0.31 | 0.004 |
| Linoleic acid | -0.19 | -0.25 | 0.09 | -0.13 | 0.40* | 0.20 | 0.11 | 0.34 | 0.34 | 0.35 | 0.36 | 0.24 |
| Linolenic acid | 0.10 | -0.07 | 0.03 | -0.19 | 0.41* | 0.28 | -0.05 | 0.35 | 0.40* | 0.41* | 0.29 | 0.25 |
| Asn | -0.14 | -0.23 | 0.07 | -0.12 | 0.51* | 0.30 | 0.03 | 0.28 | 0.16 | 0.32 | 0.33 | 0.32 |
| Trp | -0.09 | -0.19 | 0.002 | -0.24 | 0.44* | 0.21 | -0.01 | 0.27 | 0.23 | 0.41* | 0.41* | 0.26 |
| His | -0.22 | -0.29 | 0.06 | -0.22 | 0.42* | 0.18 | 0.04 | 0.29 | 0.22 | 0.40* | 0.40* | 0.24 |
| Gln | 0.02 | -0.24 | 0.14 | -0.31 | 0.21 | 0.25 | -0.01 | 0.42* | 0.26 | 0.33 | 0.30 | 0.15 |
| Raffinose | 0.01 | -0.03 | -0.21 | 0.07 | 0.20 | 0.16 | 0.12 | 0.14 | 0.40* | 0.28 | 0.10 | 0.26 |
| Xylitol | -0.21 | -0.30 | 0.25 | -0.24 | 0.02 | 0.04 | -0.12 | 0.43* | 0.15 | 0.10 | 0.17 | 0.08 |
| Mannitol | -0.15 | -0.13 | 0.46* | 0.15 | -0.23 | -0.06 | 0.15 | 0.29 | 0.09 | -0.08 | 0.01 | -0.14 |
| Inositol | -0.17 | -0.17 | -0.19 | -0.04 | -0.43* | -0.07 | 0.01 | 0.003 | 0.09 | -0.08 | -0.10 | -0.11 |
| Fru-Leu | -0.17 | -0.27 | 0.07 | 0.08 | 0.26 | 0.32 | 0.13 | 0.18 | 0.40* | 0.19 | 0.17 | 0.17 |
| Fru-IIe | -0.18 | -0.28 | 0.09 | 0.07 | 0.26 | 0.31 | 0.13 | 0.18 | 0.40* | 0.18 | 0.17 | 0.16 |
| GLU | -0.16 | -0.24 | 0.08 | 0.09 | 0.22 | 0.33 | 0.23 | 0.21 | 0.40* | 0.18 | 0.16 | 0.17 |
| Fru-Glu | -0.04 | -0.11 | -0.07 | 0.04 | 0.16 | 0.29 | 0.19 | 0.19 | 0.41* | 0.20 | 0.10 | 0.15 |
| Neochlorogenic acid | -0.04 | -0.05 | 0.70** | -0.11 | -0.17 | 0.31 | 0.03 | 0.02 | 0.24 | 0.07 | -0.15 | -0.38 |
| Chlorogenic acid | 0.09 | -0.54* | 0.21 | -0.42* | 0.30 | 0.04 | -0.02 | -0.24 | 0.13 | -0.06 | -0.07 | -0.14 |
| Cryptochlorogenic acid | 0.12 | 0.64** | -0.22 | 0.67** | -0.46* | -0.22 | 0.43* | 0.19 | -0.40* | -0.28 | -0.28 | -0.20 |
| Scopoletin | 0.57* | 0.71** | -0.37 | 0.75** | -0.07 | -0.49* | 0.55* | 0.36 | -0.31 | -0.50* | -0.26 | -0.20 |
| Rutin | 0.20 | 0.60** | -0.24 | 0.42* | -0.03 | -0.37 | 0.78* | -0.44* | -0.47* | -0.53* | -0.50 | -0.33 |

* and ** represented *P*<0.05 and *P*<0.01, respectively

**Table S18** Pearson correlation between aroma precursors and quality characteristics of cigar tobaccos from different origins of middle leaves.

| Compounds | Aroma quality | Aroma volume | Irritation | Combustibility | Gray | Aftertaste | Sweetness | Miscellaneous aroma |
| --- | --- | --- | --- | --- | --- | --- | --- | --- |
| Linoleic acid | 0.36 | 0.44* | 0.05 | 0.35 | 0.34 | 0.08 | 0.09 | 0.28 |
| Linolenic acid | 0.40* | 0.40* | 0.07 | 0.11 | 0.09 | 0.21 | 0.20 | 0.25 |
| Asn | 0.41* | 0.40* | 0.02 | 0.42* | 0.34 | 0.22 | 0.26 | 0.20 |
| His | 0.46* | 0.53* | 0.06 | 0.33 | 0.34 | 0.16 | 0.18 | 0.27 |
| Fru-Leu | 0.31 | 0.33 | 0.14 | 0.48* | 0.44* | 0.12 | 0.08 | 0.26 |
| Fru-IIe | 0.32 | 0.34 | 0.14 | 0.40* | 0.44* | 0.11 | 0.07 | 0.27 |
| GLU | 0.24 | 0.28 | 0.07 | 0.36 | 0.44* | 0.10 | 0.07 | 0.20 |
| Oxalic acid | 0.23 | 0.30 | 0.26 | 0.41* | 0.18 | 0.16 | 0.22 | 0.18 |
| Phe | 0.30 | 0.40* | -0.03 | 0.10 | 0.09 | 0.09 | 0.14 | 0.08 |
| Trp | 0.44* | 0.49* | 0.02 | 0.23 | 0.18 | 0.17 | 0.22 | 0.24 |
| Mannose | 0.25 | 0.25 | 0.15 | 0.42* | 0.48* | 0.14 | 0.12 | 0.23 |
| Fructose | 0.26 | 0.26 | 0.15 | 0.40* | 0.48* | 0.14 | 0.11 | 0.24 |
| Glucose | 0.26 | 0.26 | 0.14 | 0.43* | 0.48* | 0.14 | 0.12 | 0.23 |
| Fru-Asn | 0.35 | 0.36 | 0.15 | 0.45* | 0.48* | 0.17 | 0.13 | 0.31 |
| Fru-Phe | 0.23 | 0.22 | 0.14 | 0.30 | 0.42* | 0.15 | 0.14 | 0.22 |
| Fru-Pro | 0.20 | 0.19 | 0.12 | 0.33 | 0.43* | 0.15 | 0.13 | 0.20 |
| Fru-Trp | 0.16 | 0.14 | 0.13 | 0.27 | 0.40* | 0.15 | 0.14 | 0.15 |
| Neochlorogenic acid | 0.02 | 0.02 | -0.06 | -0.02 | 0.44* | -0.02 | -0.41* | -0.42* |
| Chlorogenic acid | -0.24 | -0.24 | 0.02 | -0.32 | -0.04 | 0.07 | 0.43* | 0.66** |
| Cryptochlorogenic acid | 0.19 | 0.19 | 0.11 | 0.26 | 0.26 | -0.37 | -0.68** | -0.51* |
| Scopoletin | 0.36 | 0.36 | 0.21 | 0.54* | -0.26 | -0.59* | -0.51* | -0.64** |
| Rutin | -0.44* | -0.44* | 0.24 | -0.61** | 0.26 | -0.22 | -0.57* | -0.58* |

* and ** represented *P*<0.05 and *P*<0.01, respectively
